# Bare Metal Stenting for Residual Arch Dissections: A Computational Analysis

**DOI:** 10.1007/s13239-025-00799-6

**Published:** 2025-08-04

**Authors:** Žiga Donik, Sanjeev Dhara, Willa Li, Blessing Nnate, Seth Sankary, Kayla Polcari, Mary Alyssa Varsanik, Kameel Khabaz, Ross Milner, Nhung Nguyen, Janez Kramberger, Luka Pocivavsek

**Affiliations:** 1 Faculty of Mechanical Engineering, University of Maribor, Maribor, Slovenia; 2 Section of Vascular Surgery and Endovascular Therapy, Department of Surgery, The University of Chicago, Chicago, IL USA; 3 Division of Vascular Surgery, Department of Surgery, Emory University, Atlanta, GA USA

**Keywords:** Bare metal stent, Type A thoracic aortic dissection, Finite element analysis, Computational fluid dynamics

## Abstract

**Purpose:**

Type A Thoracic Aortic Dissections are a highly morbid and complex clinical challenge often managed with hemiarch or total arch repair. Hemiarch repair is more commonly performed due to improved neurologic morbidity profile however it leaves behind a residual dissection flap which can lead to aneurysmal degeneration. Bare metal stent placement in conjunction with hemiarch repair is a novel technique which can theoretically avoid leaving a residual dissection flap. In this paper we analyze the biomechanical changes observed after in silico deployment of a bare metal stent in a post-hemiarch type A aortic dissection.

**Methods:**

We obtain computed tomography scans from pre-operative bare metal stent patients and perform high-fidelity segmentations. This geometry is then utilized for in silico stent deployment via finite element analysis. Deformed geometries are then utilized for computational fluid dynamic simulations to analyze the evolution of pressure gradients in the aorta.

**Results:**

We analyze the resulting geometry from in silico stent deployment for three different stiffness ratios between the flap and aortic wall. We demonstrate an acceptable stress evolution in the stent across all 3 stiffness configurations. We show a reduction in the false luminal volume across all stiffness ratios. Our analysis of pressure distributions that evolve in the aorta show that even in scenarios of high flap stiffness, where the false lumen volume shrinks correspondingly less, we still achieve a reduction in the pressure gradient across the aorta.

**Conclusion:**

We show that bare metal stent deployment hemodynamically stabilizes the aorta via our finite element analysis and subsequent computational fluid dynamic modelling.

**Supplementary Information:**

The online version contains supplementary material available at 10.1007/s13239-025-00799-6.

## Introduction

Type A Thoracic Aortic Dissection is a highly morbid condition and deemed a surgical emergency with notable mortality rates at 0.5% per hour versus 0.09% for those managed non-operatively versus operatively, respectively [1]. Surgical management strategies include hemiarch repair, total arch repair with elephant trunk/frozen elephant trunk, or newer approaches incorporating endovascular technologies. The current treatment options can be thought of in a paradigm ranging from hemiarch repair to total arch repair (TAR) with regards to the benefits and drawbacks. Hemiarch repair resects the primary proximal entry tear and replaces the ascending aorta with a tube graft. Although believed of as having lower morbidity and mortality (particularly neurologic complications such as stroke or spinal cord ischemia) it often leaves a persistent distal false lumen which is associated with deleterious future consequences such as need for re-operation or aneurysmal degeneration [2]. Rylski et al. examined the fate of patients with a residual dissection after hemiarch repair and determined a continued aortic growth rate of 1.5 mm/year in these patients, with > 30% requiring a thoracic aortic re-operation distal to the hemiarch repair [3]. The majority of patients with rapid aortic growth have been found to have distal re-entry tears [4]. Sealing the distal dissection in the descending aorta to prevent aneurysmal degeneration following ascending aortic repair remains an operative challenge. Solutions to this problem bring us to the other end of the spectrum of type A aortic dissection management. TAR with elephant trunk (ET)/frozen elephant trunk (FET) carries the advantage of replacing the entire arch and helps to facilitate repair of distal segments of the thoracic aorta. However, it is a more involved operation and is thought to carry more significant neurologic risks. An analysis by Kim et al. found a roughly 40% increase in the freedom from death and neurologic injury at long-term follow up in those who underwent hemiarch repair as compared to total arch repair [5]. As such, there remains an opening for approaches which manage the residual dissection flap created by the hemiarch technique without the added mortality/morbidity risk of TAR, leading to the development of techniques such as total endovascular repair or hybrid approaches (such as the Ascyrus Medical Dissection Stent and bare metal stenting (BMS) using the Zenith Dissection Endovascular Stent (ZDES, Cook Medical)) [6–8].

The AMDS stent is composed of a partially covered aortic arch hybrid prosthesis made of Teflon fabric with an uncovered attached nitinol stent which can be deployed into the true lumen (TL) at the time of the repair. This modality has been shown to have impressive rates of true lumen expansion and stability with partial to complete false lumen (FL) thrombosis thereby abrogating aneurysmal degeneration risks, while maintaining an impressive safety profile as it relates to adverse neurologic events and mortality [6, 9]. Alternatively, totally endovascular approaches such as the Nexus Aortic Arch Stent Graft system have been trialed showing favorable results with regards to mortality and avoidance of adverse neurologic events, however trials involving this device did not include acute dissection patients [7]. Regardless there have been recent papers arguing for the feasibility of total endovascular repair in type A dissections [10].

BMS of the residual dissection flap to increase the true lumen size and result in positive aortic remodeling has also been trialed as a hybrid option [8]. The STABLE I and II trials studied the endovascular procedure called Provisional Extension To Induce Complete Attachment (PETTICOAT) which utilized the Cook Zenith Dissection Endovascular System to induce positive aortic remodeling with minimal rates of spinal cord ischemia in patients with Type B Thoracic Aortic Dissection [11, 12]. The results showed that at 5-years, in all groups, the majority of patients had thrombosis of the FL and stabilization of aneurysmal degeneration in both the thoracic (stent graft) region and abdominal (BMS) region of the aorta. Endicott et al*.* recently published a case-series where they expanded the PETTICOAT concept to the aortic arch thereby staging the arch stabilization in type A dissections compared to AMDS which must be implanted during the index type A repair [8].

While the concept of dissection flap stabilization and re-attachment is gaining clinical traction as shown by the above studies, there lacks a mechanistic understanding of the biomechanical principles which drive aortic remodeling post-BMS placement into a dissected aorta. A growing body of research has employed computational modeling techniques to simulate thoracic endovascular aortic repair (TEVAR), focusing on both the mechanical deployment of stent grafts and associated hemodynamics. Studies have utilized finite element analysis (FEA) to simulate stent graft (SG) implantation, capturing the device–artery interaction under physiological conditions [13–26]. These works vary in terms of anatomical modeling from idealized aortas [18, 21, 25] to patient-specific geometries derived from CTA imaging [13–17, 19–22, 26] and include both rigid and deformable vessel representations. Some studies also incorporate arterial pre-stress to mimic realistic physiological conditions [20, 23]. Early simulation efforts, such as those by De Bock et al. [27] demonstrated the feasibility of virtual stenting using simplified abdominal models validated against experimental data. Subsequent studies advanced this framework, including patient-specific thoracic simulations by Auricchio et al. [13] and compliant arterial models with morphing deployment methods by Perrin et al. [28] More recent work has explored key clinical factors like oversizing ratios [15, 19], and model verification against post-operative imaging or benchtop data [20, 22, 25].

In this paper, utilizing computed tomography (CT) imaging, we create a patient-specific in silico model of an aorta after hemiarch repair. Utilizing FEA as well as computational fluid dynamic (CFD) analysis we then simulate the deployment of a bare metal stent to investigate the resulting stress distribution, evolution of true lumen geometry, and the evolving pressure differential between the true and false lumens. We demonstrate a mechanistic justification for the efficacy of BMS as a management strategy for residual dissection flaps and persistent distal false lumens after hemiarch repair of Type A dissections. Our study contributes to the evolving field of in-silico* thoracic aortic surgery* by modeling bare metal stent (BMS) deployment in dissected thoracic aortas, with attention to the interaction between the device and a variably stiff intimal flap. This is the first ever simulation of the novel modified aortic arch PETTICOAT technique.

## Methods

The authors have no conflicts of interest to disclose. This work has been approved by the University of Chicago Institutional Review Board and adheres to the Declaration of Helsinki.

### Segmentation

We obtained a pre-operative CT scan on a 71-year-old female patient who was to undergo bare metal stenting. The patient had a recent history of a type A dissection with an already completed hemiarch repair. The patient was not a candidate for TAR and we were able to utilize the BMS for compassionate use. The CT scan was obtained and then utilizing a semi-automated segmentation procedure previously described within our group, we were able to reconstruct high-fidelity 3D representations of the aortic geometry including both the aortic wall as well as the dissection flap [29, 30]. Briefly, the outline of the entire aorta is first mapped by an automated algorithm which is then manually reviewed for accuracy. Through a combination of dilation and smoothing functions we can then recreate a smooth aortic surface for FEA. The flap is often not visible on CT scans due to resolution limitations. We can however clearly define the true and false lumens allowing us to take the region between them as the flap. Using Boolean operations to subtract the true and false lumen from the solid model of the aorta, we only get the aortic wall and the dissection flap, which were then meshed with 214658 tetrahedral solid elements.

### Finite Element Analysis

**Fig. 1:**
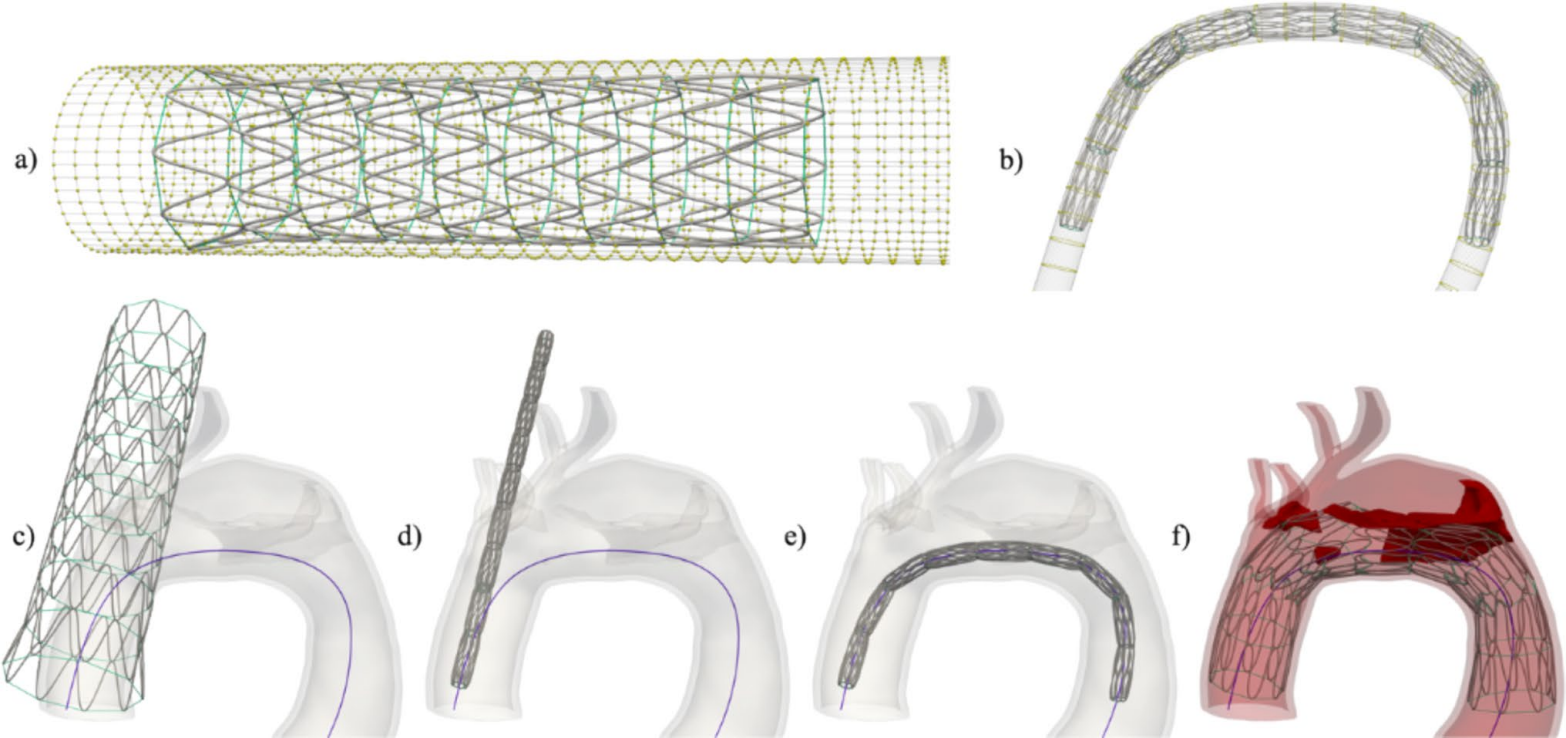
A crimping tool is used to simulate stent deployment in several steps. The crimper is placed along the stent centerline, wrapping the stent (**a**). Each crimper node (yellow point) is assigned a path in each coordinate axis direction to achieve the desired deformation of the stent. The crimper and subsequently the stent have to be properly aligned with the true lumen centerline (**b**). In the first step, the stent is radially compressed to fit inside the narrowest part of the lumen (**c**, **d**). The stent is then gradually bent until it is aligned with the true lumen centerline, shown in blue (**d**, **e**). Finally, the aorta is added to the simulation and the stent self-expands when the diameter of the crimper is increased and the stent to crimper contact is deactivated. In the first two steps (**c**–**e**), the aorta is not a part of the simulation, which is highlighted by the transparency

Finite element analysis is performed on the computational models of the patient-specific aorta and the bare metal stent, which is virtually deployed inside the aortic arch. FEA was done using LS-DYNA and input files were generated using Python and Gmsh. Beam and solid finite elements were used to model the stent wire and artery, respectively. Our bare metal stent was modelled on the Zenith Dissection Endovascular Stent by Cook Medical. The stent is 185 mm long and comprised of 9 support rings. We created a finite element mesh based on the design of the stent with linear Hughes-Liu beam elements with cross section integration representing metal wire. The finite element mesh with 8100 beam elements was chosen based on the previously performed mesh convergence study. The sutures along the model were simulated using discrete spring elements with negligible stiffness in compression. A sinusoidal function was utilized to model each of the rings. The model was created in a 2D plane and then wrapped into a cylinder to create the final stent. Figure 1a shows our model.

The deployment of the stent is modeled to follow the procedural steps typically performed during surgical intervention. Initially, the stent is compressed to a smaller diameter and registered to align with the centerline of the true lumen, as shown in Fig. 1b. A cylindrical device, referred to as the crimper, is used to facilitate stent manipulation and deployment. The crimper is a tubular shell structure composed of 2,350 quadrilateral elements, visible in Fig. 1a, which fully encloses the stent. Its function is not to actively expand the stent, but to provide controlled deformation through predefined nodal displacements.

In the first deployment step, the proximal end of the stent is fixed (Fig. 1c) and the crimper is radially contracted to compress the stent to the minimum required diameter—equivalent to the smallest inscribed sphere fitting within the narrowest segment of the true lumen (Fig. 1d). This proximal fixation mimics intra-operative handling of the device during initial positioning.

**Fig. 2:**
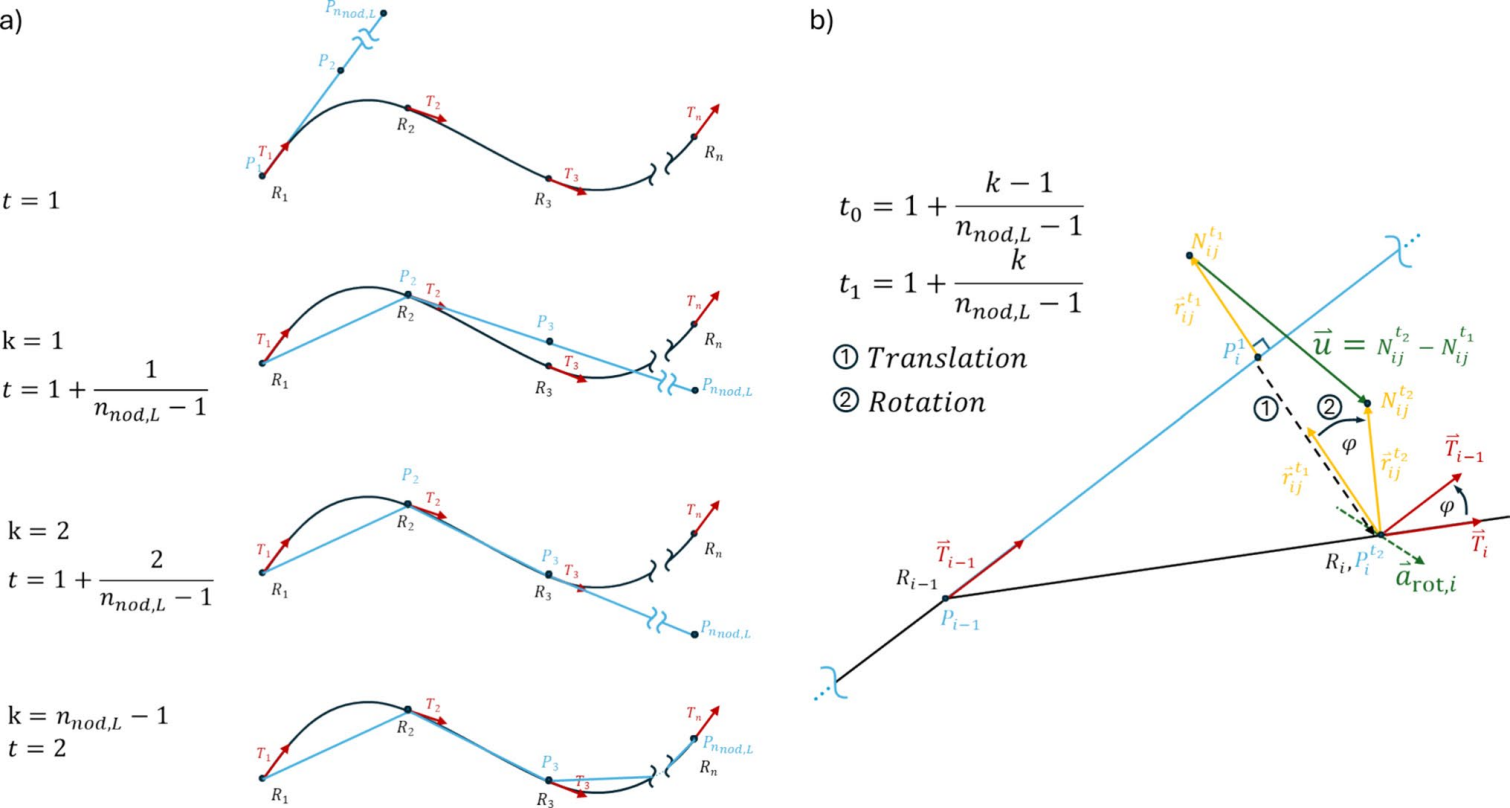
The transformations to align the crimper to the centerline of the aorta and compute the crimper nodal coordinates throughout the second simulation step to achieve the desired motion. a) The alignment of the crimper centerline $${C}_{c}$$ (blue) with the vessel centerline $${C}_{0}$$ (black) at different intermediate positions $$k$$ following $$P_{i}^{t} = \left\{ {\begin{array}{*{20}c} {R_{i} } & {;i \le k} \\ {P_{k} + \overset{\lower0.5em\hbox{$\smash{\scriptscriptstyle\rightharpoonup}$}}{T} _{k} \cdot \sum _{{m = k}}^{i} \left| {P_{{m + 1}} - P_{m} } \right|} & {;i > k} \\ \end{array} } \right.$$, where $$t=1+\frac{k}{{n}_{\text{nod},\text{L}}-1}$$ and $$k=1\dots \left({n}_{\text{nod},\text{L}}-1\right)$$. The vessel centerline tangents are shown as red vectors. Each line consists of $${n}_{\text{nod},\text{L}}$$ points, but only a part of the line is shown. b) The transformation applied to the nodes in the state $${t}_{0}$$ to obtain the coordinates of the crimper nodes at the intermediate state $${t}_{1}$$. A translation corresponding to the points on the centerlines is first applied, then the rotation about the axis $$\overset{\lower0.5em\hbox{$\smash{\scriptscriptstyle\rightharpoonup}$}}{a} _{{{\text{rot}},i}}$$ by the angle $${\varphi }_{i}$$ is added to finalize the intermediate position of the node

Next, the crimper is gradually aligned along the aortic centerline using a point-by-point transformation (Figs. 1e, 2), calculated via a Python script. Each node on the crimper is assigned a predefined spatial path to follow, which ensures that the cylindrical crimper conforms precisely to the curvature and orientation of the patient-specific aortic centerline while maintaining the crimper’s structural coherence and cylindrical shape. This alignment ensures accurate stent tracking with respect to vessel curvature and geometry.

Once fully positioned, the crimper is radially expanded. This does not directly push the stent outward but rather relieves the external constraint and permits the self-expanding stent to deploy passively, driven by its own stored elastic energy. This controlled release mitigates abrupt contact interactions and improves simulation stability. When the crimper exceeds the diameter of the aortic lumen, contact with the stent is deactivated, allowing it to interact solely with the vessel wall (Fig. 1f).

**Fig. 3:**
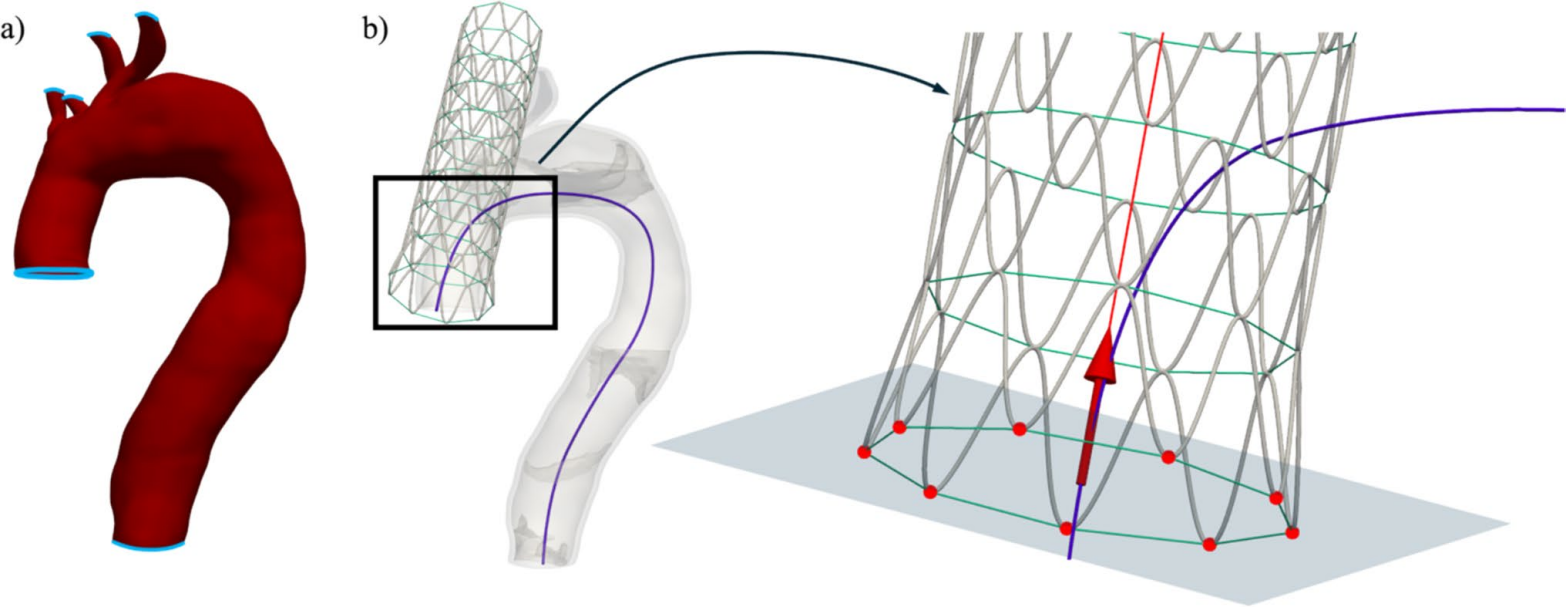
Boundary conditions on the aorta (**a**) and stent (**b**). The aorta is fixed at the proximal and distal end, as well as at the branch vessels. The stent is positioned on the true lumen centerline (dark blue line) such that the stent centerline (red line) is aligned with the tangent vector (red arrow) at the chosen point of deployment on the true lumen centerline. The endpoints of the stent (red dots) are assigned a translational constraint in the direction of the true lumen centerline tangent vector (red arrow), meaning that the points (nodes) are allowed to move freely along the plane (blue) normal to the tangent vector (red arrow)

The aorta is fixed in all directions at the proximal and distal cut-off as well as at the edge of the arch branches, as shown in Fig. 3a. The stent is placed along the centerline tangent, 14 mm from the aortic root. This location corresponds to its intra-operative placement. The peaks of the first stent ring are constrained in the translational direction of the centerline tangent to keep the proximal end of the stent in place and allow movement only in the plane, perpendicular to the centerline tangent (see Fig. 3b).

In implicit simulations, the mortar contact (MORTAR option) is the preferred type of contact that effectively encompasses all interactions between different element types. One contact definition involves the stent and the crimper, while another includes the stent and the artery. As the dissection flap is a separate object, another two contact pairs are required. Those include the flap to artery and the stent to flap contact. Notably, no contact is defined between the crimper and the vessel wall. During crimping, positioning, and expansion phases, the stent/crimper contact is activated, while the stent-artery contact remains inactive until the final step of stent expansion. A friction coefficient of 0.1 is applied to all defined contacts.

The metal support rings of the stent are made of nitinol and modelled as linear elastic model with Young’s modulus of 83 GPa, Poisson’s ratio of 0.33, and density of 6450 kg/m [3]. Although Nitinol is known for its superelastic behavior due to stress-induced phase transformation, a linear elastic approximation was employed here based on the relatively small strain levels observed during simulation. These strain levels remained close to the upper bound of the linear elastic regime for Nitinol, which is approximately 1% [31, 32]. As the focus of this study is on deployment mechanics and final stent configuration, the linear elastic model was considered sufficient.

The crimping tool is steel and simulated as a linear elastic model with Young’s modulus of 210 GPa, Poisson’s ratio of 0.3, and density of 7850 kg/m [3]. The mechanical behavior of the aortic wall is influenced by different distributions of collagen fibers and exhibits anisotropy. Given the morphological complexity of aortic dissection and uncertainties regarding fiber orientation and properties of the dissection membrane, an isotropic hyperelastic material model proposed by Vorp and Raghavan was used. The values of material constants ${C}_{10}=0.174 \text{MPa}$ and ${C}_{20}=1.881 \text{MPa}$ were acquired from the literature [33]. As a separate element set, the dissection flap is prescribed the same material, but three different intimal flap stiffnesses are examined. The different dissection flap stiffness ratios represent the chronicity of the dissection. In acute AD, the intimal flap stiffness is lower, compared to the aortic wall, whereas with chronic AD, the stiffness of the dissection flap increases [34]. To model this evolution of flap stiffness with chronicity, the flap material stiffness is scaled by 1, 0.1, and 0.01 by scaling the material constants ${C}_{10}$ and ${C}_{20}$, presented in Table 1. The utilized flap stiffness values are not patient-specific and were selected for a parametric sensitivity study to capture the potential range of flap stiffness seen in acute versus chronic dissections. Literature suggests that stiffness can vary by orders of magnitude depending on tissue remodeling and wall layer [34–36]. The lowest flap stiffness case (0.01:1) was also included to explore extreme flap mobility and its impact on stent interaction and flow, which is relevant for follow-up CFD analysis.

**Table 1:** The material parameters of the Vorp-Raghavan material model for the aortic wall and the dissection flap

	${C}_{10} /\text{MPa}$	${C}_{20} /\text{MPa}$
Aortic wall	0.174	1.881
Dissection flap at 1:1 stiffness ratio	0.174	1.881
Dissection flap at 1:0.1 stiffness ratio	0.0174	0.1881
Dissection flap at 1:0.01 stiffness ratio	0.00174	0.01881

Three distinct configurations with different dissection membrane stiffnesses are studied by scaling the stiffness of the aorta by 1, 0.1, and 0.01 to obtain the properties of the flap

### Radial Force Calculation

We quantified the stent’s radial force during crimping, corresponding to the first deployment step shown in Fig. 1c and d. The nodal reaction force data from the crimper was used to estimate the radial force which was then normalized per unit length. To isolate the stent’s contribution, we ran two additional simulations: (A) with both stent and crimper, and (B) with the crimper alone. Subtracting the reaction force in simulation B from A yielded the force acting solely on the stent, enabling estimation of the radial force.

### Computational Fluid Mechanic Analysis

In conjunction with the FEA, CFD simulations were conducted to examine the flow conditions within the aorta in different configurations. A mesh independence study was performed. Four different mesh discretization sizes were chosen: 0.00075 m, 0.000625 m, 0.0005 m, and 0.000375 m. These resulted in four different mesh sizes: 682721, 1178688, 2301504, and 5456424 elements. The three largest mesh sizes demonstrated adequate convergence. Figures S1 and S2 show the velocity and pressure distributions at the three largest mesh sizes. The discretization size of 0.0005 m (2301504 mesh elements) was chosen to optimize for computational cost. For the CFD analysis, four distinct models were utilized. Firstly, the non-stented patient-specific aorta served as the baseline for comparing flow dynamics with the stented aorta. Additionally, three variations of the stented aorta were simulated, each with a different stiffness ratio of the dissection flap compared to the aorta: 1:1, 1:0.1, and 1:0.01. These ratios represented varying degrees of intimal flap stiffness, influencing stent deployment and, subsequently, the flow patterns within the aorta.

The deformed model of the aorta, resulting from the FEA of stent deployment, is exported as an STL file. This is used as the input geometry for the aortic wall and flap. These models were inputted as 3D STL mesh geometries into XFLOW, a commercial CFD software employing the Lattice-Boltzmann method. A rigid-wall model is assumed. The boundary conditions are defined at the aorta inlet (at the level of the coronary artery takeoff from the aortic root), aorta outlet (at the level of the celiac artery takeoff), and aortic arch branch vessels. At the inlet, a velocity boundary condition is applied, with the aorta inlet velocity defined by an oscillating function to replicate cardiac systole and diastole using the following equation: [37].

$${v}_{\text{inlet}}\left(t\right)=0.4248 - 0.06805\cdot \text{cos}\left(\frac{14.77}{2}\cdot t\right)+ 0.1644 \cdot \text{sin}\left(\frac{14.77}{2}\cdot t\right) - 0.08076 \cdot \text{cos}\left(2 \cdot \frac{14.77}{2}\cdot t\right)- 0.00885 \cdot \text{sin}\left(2 \cdot \frac{14.77}{2}\cdot t\right) - 0.04299 \cdot \text{cos}\left(3 \cdot \frac{14.77}{2}\cdot t\right)- 0.0357 \cdot \text{sin}\left(3 \cdot \frac{14.77}{2}\cdot t\right) + 0.01435 \cdot \text{cos}\left(4 \cdot \frac{14.77}{2}\cdot t\right)- 0.02632 \cdot \text{sin}\left(4 \cdot \frac{14.77}{2}\cdot t\right) + 0.01557 \cdot \text{cos}\left(5 \cdot \frac{14.77}{2}\cdot t\right)- 0.005171 \cdot sin(5 \cdot \frac{14.77}{2} \cdot t)$$

Two different sets of pressure boundary conditions were applied. A uniform pressure boundary condition was applied to the aorta outlet and the arch branch vessels, with the pressure set to 100 mmHg, replicating approximate mean physiological blood pressure. To study the impact of changing the pressure gradient we also ran a separate set of simulations with a varying pressure gradient utilizing an oscillating function to replicate the cardiac cycle like the velocity inlet. The oscillating function was tuned to 120/80 mmHg, replicating physiologic blood pressure. The equation can be seen below:

$${Pressure}_{\text{inlet}}\left(t\right)=12564.24 - 749.36\cdot \text{cos}\left(\frac{14.77}{2}\cdot t\right)+ 1810.36 \cdot \text{sin}\left(\frac{14.77}{2}\cdot t\right) - 889.32 \cdot \text{cos}\left(2 \cdot \frac{14.77}{2}\cdot t\right)- 97.46 \cdot \text{sin}\left(2 \cdot \frac{14.77}{2}\cdot t\right) - 473.40 \cdot \text{cos}\left(3 \cdot \frac{14.77}{2}\cdot t\right)- 393.13 \cdot \text{sin}\left(3 \cdot \frac{14.77}{2}\cdot t\right) + 158.02 \cdot \text{cos}\left(4 \cdot \frac{14.77}{2}\cdot t\right)- 289.83 \cdot \text{sin}\left(4 \cdot \frac{14.77}{2}\cdot t\right) + 171.46 \cdot \text{cos}\left(5 \cdot \frac{14.77}{2}\cdot t\right)- 56.94\cdot sin(5 \cdot \frac{14.77}{2} \cdot t)$$

**Fig. 4:**
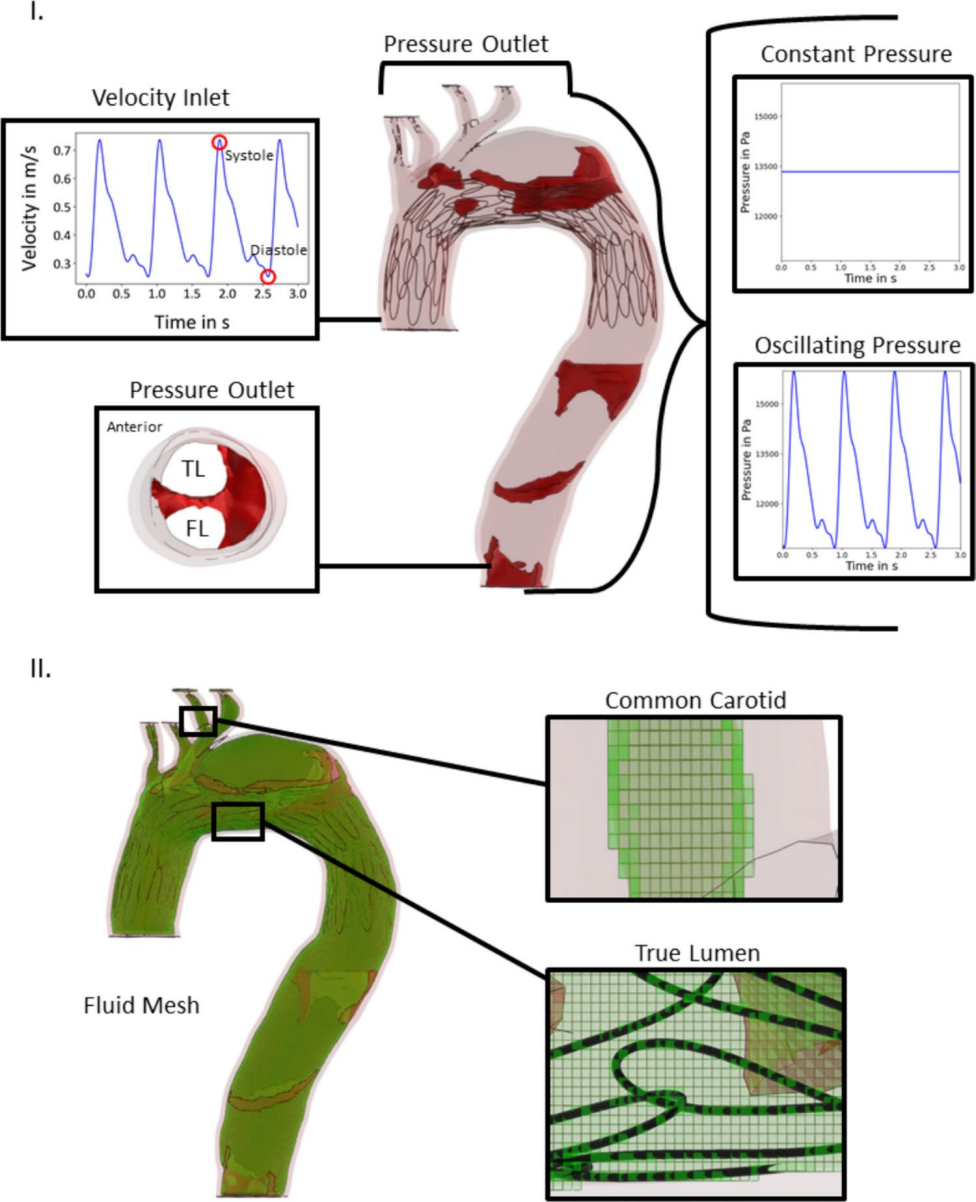
The boundary conditions (**A**) and the lattice fluid mesh (**B**) used in the CFD simulation. The inlet velocity is defined at the proximal end of the ascending aorta by a function resembling a cardiac cycle. The outlets at the aortic arch branch vessels and at the distal end of the descending aorta are prescribed an outlet pressure of 100 mmHg to resemble mean physiological blood pressure. Additional simulations were run with a varying pressure outlet corresponding to 120/80 mmHg

The boundary conditions are shown in Fig. 4. The simulation encompasses three cardiac cycles, and the resulting data, including surface pressure, static pressure, and velocity, is exported from the software. The third cycle of CFD is utilized for further data processing. This information is transferred into Paraview, an open-source visualization software. Within Paraview, cross-sections of the bulk fluid are defined at varying points (planes) along the aorta and branch vessels. Within each cross-section, velocity and static pressure values are extracted and averaged for analysis.

## Results

**Fig. 5:**
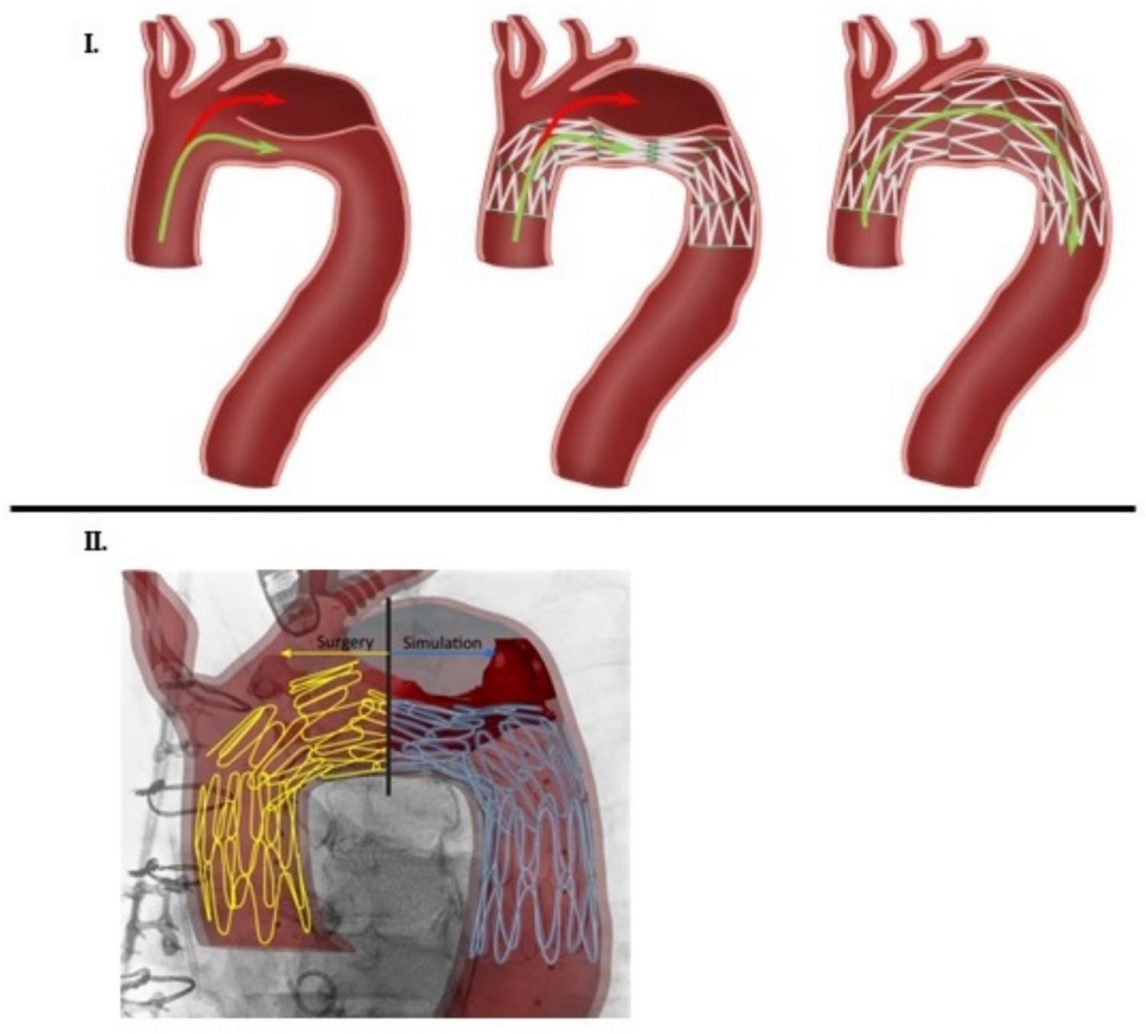
**I** Schematic drawing of aortic dissection pre- and post-bare metal stent (BMS) placement: **a** Flow in a dissected arch is diverted into both the true (green arrow) and false (red arrow) lumens. Flow into the false lumen (FL) is linked to aneurysmal dilation and aortic degeneration. **b** Deployment of the BMS in true lumen (TL) across the arch changes the flow dynamics between TL and FL, decreasing the pressure within the FL. **c** Over time, as the BMS expands through the remodeling of the intimal dissection flap, the FL is obliterated, and flow occurs only through the TL. **II** The qualitative comparison of the deformation of the stent during surgery (left, yellow) and the computational model of stent deployment (right, blue). The simulation results are superimposed on the image acquired during the surgical procedure. The qualitative visual inspection suggests a close resemblance of the stent deformation pattern to the image, which warrants the further use of the computational model

**Fig. 6:**
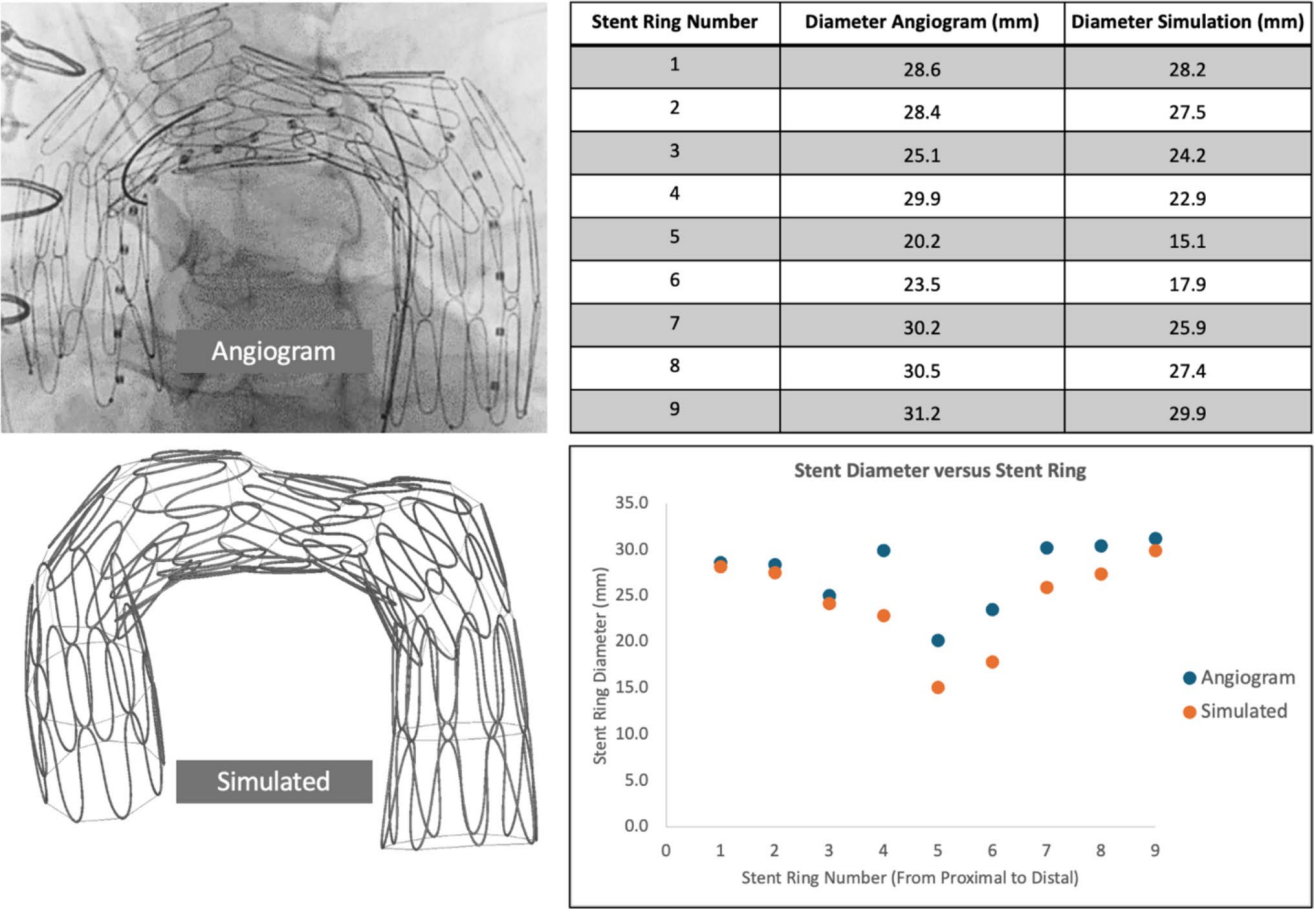
Demonstrates an image from angiogram of our BMS accompanied by a simulated representation of the BMS. Information regarding the diameter of each stent ring between the angiogram and simulated data is also given

Prior to gaining any meaningful mechanical insights regarding aortic remodeling with BMS, we verify the fidelity of in silico stent deployment versus in vivo. Figure 5 overlays our simulation of the stent deployment with an image acquired during the surgery. The left side of the image highlights the stent struts in yellow as visualized during the surgery while the right side shows our computational model of the stent in blue. Visual examination alone suggests fidelity of our model with the in vivo behavior of the bare metal stent as it is deployed in the thoracic aorta. Figure 6 provides further validation regarding the fidelity of our model as compared to the angiogram. Our model is able to capture the narrowing of the stent in the middle region (stent rings 4–7) like the angiogram.

### Finite Element Analysis

**Fig. 7:**
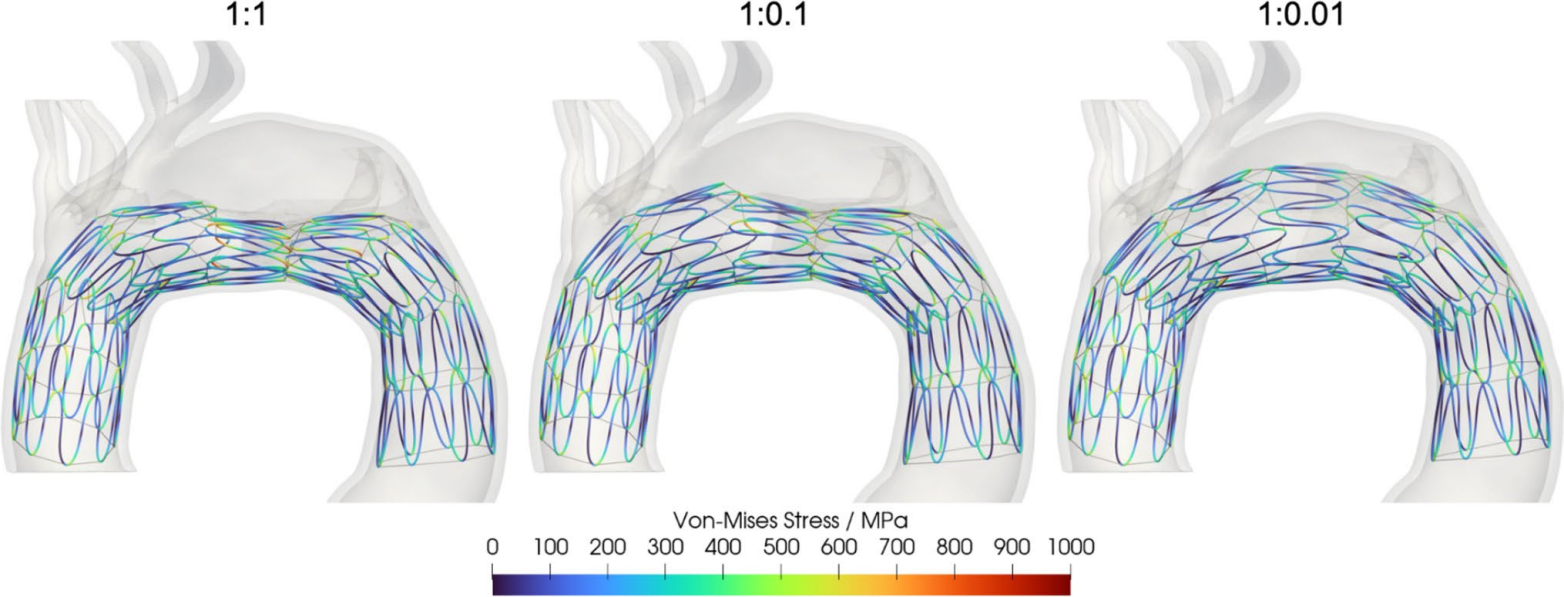
Von-Mises stress distribution in the stent after deployment for different flap stiffness ratios (1:1, 1:0.1, and 1:0.01 from left to right). Stress concentrations are evident at the peaks, but none exceeding 1000 MPa

The BMS is deployed in silico while varying the stiffness of the flap as compared to the aortic wall. We first examine the impact of the evolving stiffness on the stress distribution within the stent. Given that the Zenith Dissection Endovascular Stent (Cook Medical, Bloomington, IN, USA) is meant for deployment in the thoracoabdominal aorta, we were interested in examining the impact of the curved proximal thoracic aorta on the stent. Figure 7 shows the Von-Mises stress distribution within the stent struts after deployment with varying flap-to-aorta stiffness ratios. Stress concentration primarily appears at the peaks of the stent, where the structure bends during compression. Higher stress levels are evident in stents with higher flap stiffness due to the stent’s constrained ability to return to its initial state, leading to increased stress within the structure. Despite these variations, it is important to note that the reported ultimate tensile strength of nitinol, which stands at approximately 1000 MPa, is not reached in any configuration. These data suggest that the stent is not adversely impacted and able to withstand the angulation present at the aortic arch.

**Fig. 8:**
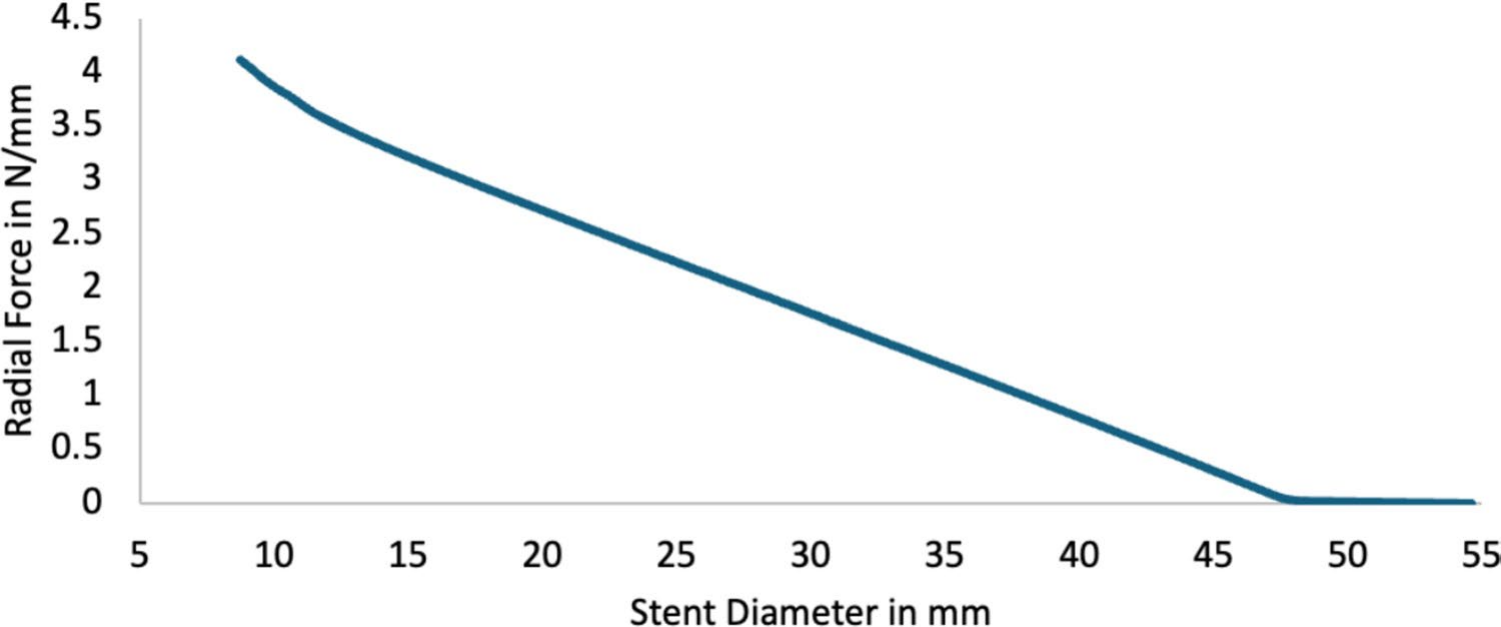
Radial Force in N/mm versus Stent Diameter in mm

**Fig. 9:**
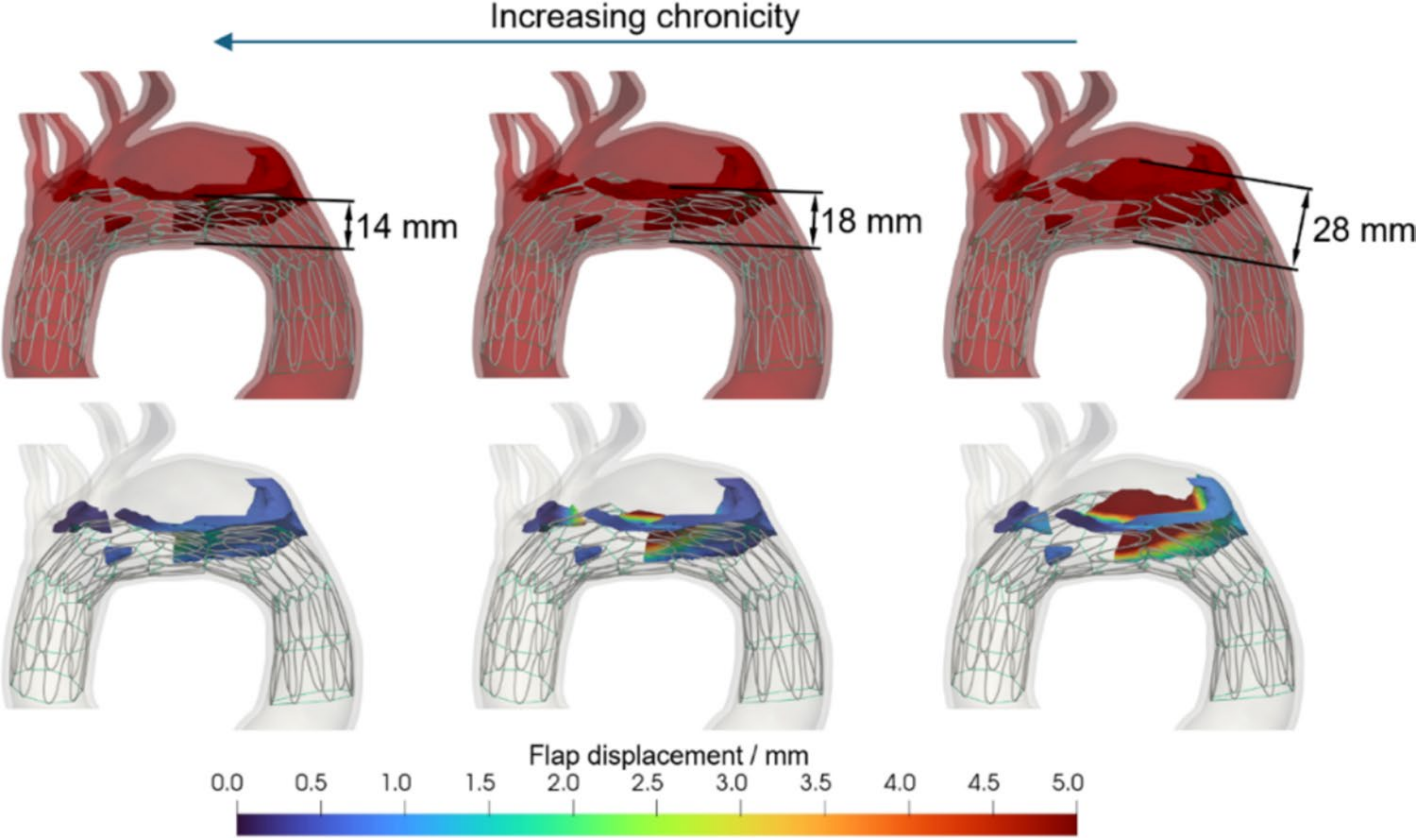
The deployed Zenith Dissection Endovascular Stent inside a patient-specific aorta with residual arch dissection. The resulting diameter of the true lumen at the narrowest part (top half) and intimal flap deflection (bottom half) are shown for different aorta-to-flap stiffness ratios (1:1, 1:0.1, and 1:0.01)

**Fig. 10:**
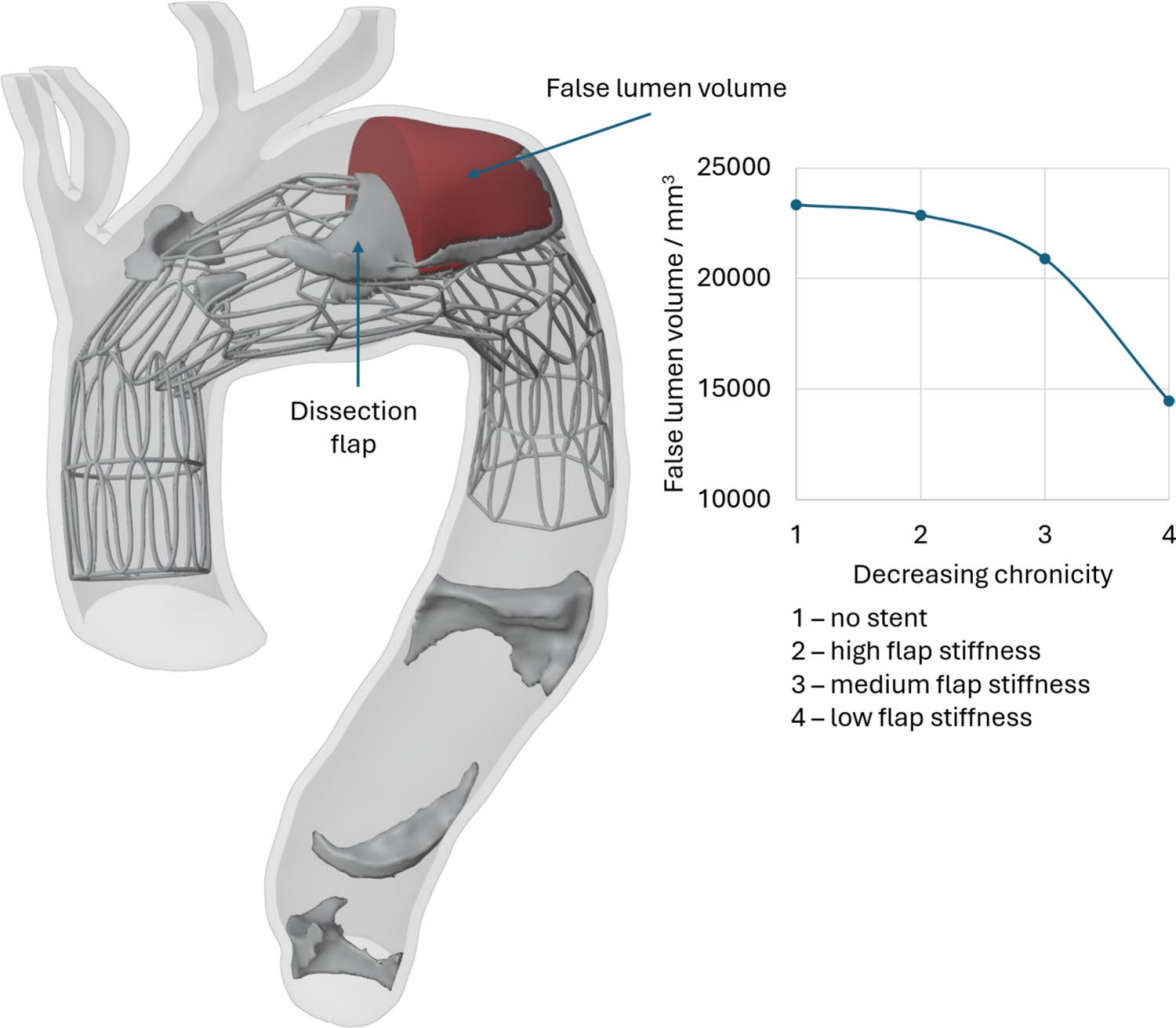
False lumen volume measured as a function of decreasing flap-to-aorta stiffness

Figure 8 reports the radial force (N/mm) versus the stent diameter. At a 30 mm diameter, our stent has a calculated radial force of 1.7 N/mm. Figure 9 demonstrates the impact of stent deployment on true lumen expansion, quantified both with resulting true lumen diameter as well as flap displacement. As hypothesized, decreasing flap-to-aorta stiffness allows for more profound true lumen expansion and more dramatic flap displacement. Notably, full intimal flap apposition to the aortic wall was not achieved even with the lowest aortic-to-flap stiffness ratio. The false lumen volume after deployment was also quantified as seen in Fig. 10. A more deformable flap in acute dissections is more responsive to stenting and moves further towards the aortic wall, displaying almost 40% decrease in false lumen volume compared to non-stented aorta.

### Computational Fluid Dynamics

**Fig. 11:**
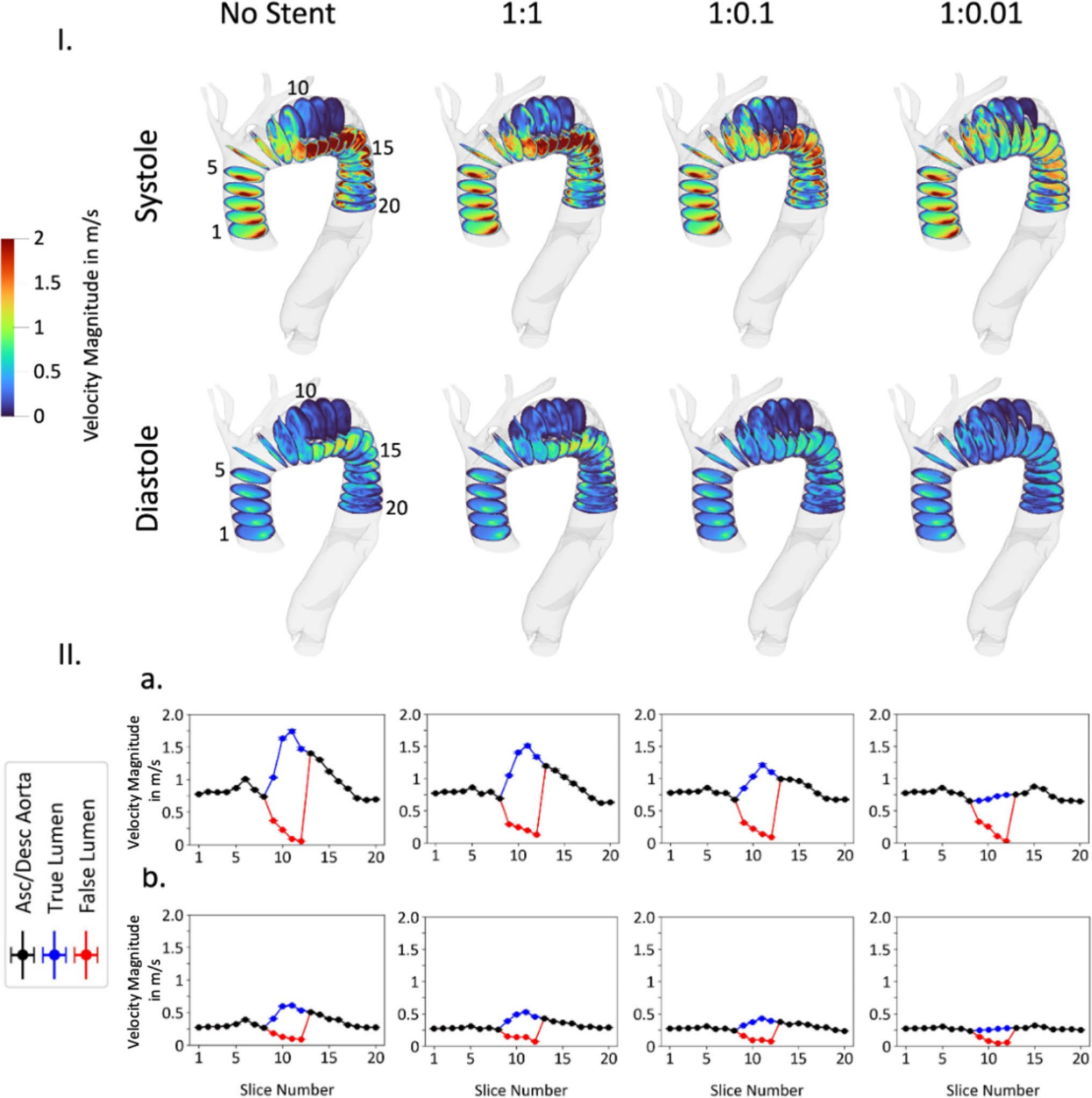
Fluid velocity throughout the aorta in non-stented and three different flap stiffness configurations (1:1, 1:0.1, and 1:0.01). The bulk fluid velocity is shown in 20 slices along the lumen, where true and false lumen are separated (**I**). The data for each slice is averaged and displayed in bottom graphs (**II**). Systole (**a**) and diastole (**b**) are presented separately

The blood flow velocity throughout the aorta is shown in Fig. 11. The simulations shown in Fig. 11 all had a constant pressure boundary condition. Increased velocity through the true lumen can be observed for the non-stented configuration, visible in both systole and diastole graphs. A minor decrease of velocity (blue curve) can be observed in the stented 1:1 configuration, while a further reduction in velocity can be observed for lower flap stiffnesses (1:0.1 and 1:0.01 stiffness ratios) due to the larger luminal area of the true lumen. The velocity in the configuration with the flap stiffness ratio of 1:0.01 stabilizes and exhibits an almost flat profile throughout the aorta.

**Fig. 12:**
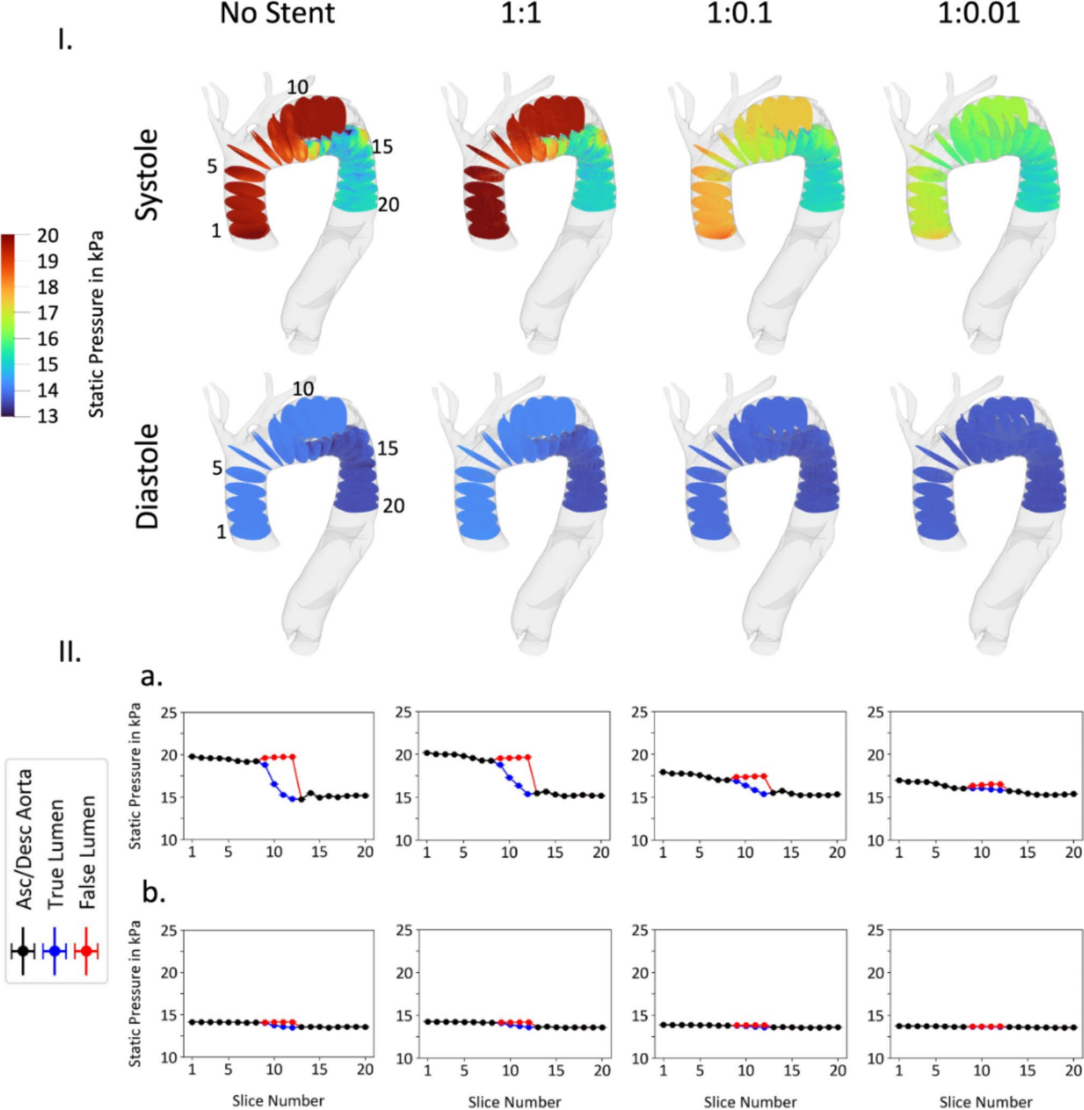
Static pressure throughout the aorta in non-stented and three different flap stiffness configurations (1:1, 1:0.1, and 1:0.01). The static pressure is shown in 20 slices along the lumen, where true and false lumen are separated (**I**). The data for each slice is averaged and displayed in bottom graphs (**II**). Systole (**a**) and diastole (**b**) are presented separately

The static pressure across the aorta with differing flap stiffness ratios is shown in Fig. 12. Decreasing flap stiffness results in more significant deflection of the flap towards the aortic wall thereby depressurizing the false lumen in systole. As seen in Fig. 12, a more pliable flap results in a lower fluctuation in pressure along the aorta while also resulting in depressurization of the false lumen. Furthermore, Fig. 12 demonstrates that increasing flap pliability in the simulation with associated true lumen expansion decreases the pressures experienced by the portions of the aorta proximal to the start of the dissection.

**Fig. 13:**
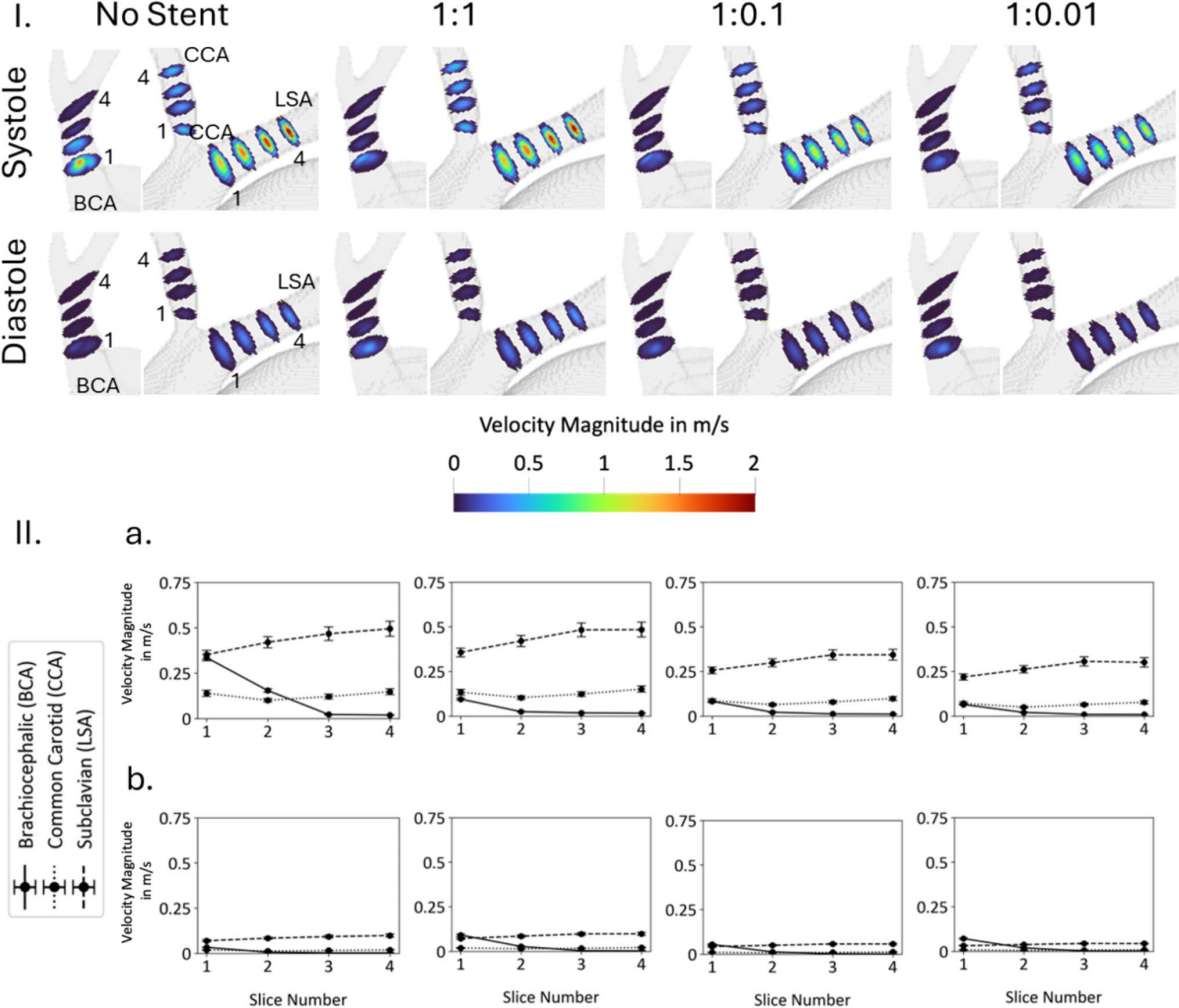
Fluid velocity throughout the aortic arch branch vessels in non-stented and three different flap stiffness configurations (1:1, 1:0.1, and 1:0.01). The bulk fluid velocity is shown in 4 slices along the brachiocephalic (BCA), common carotid (CCA), and left subclavian (LSA) arteries (**I**). The data for each slice is averaged and displayed in bottom graphs (**II**). Systole (**a**) and diastole (**b**) are presented separately

The velocity of the bulk flow inside the aortic arch branch vessels is examined. Four slices are created in the brachiocephalic, left common carotid, and left subclavian (LSA) arteries, as shown in Fig. 13. As the flap stiffness decreases, the velocity profiles become uniformly distributed across each slice and the differences between velocity magnitudes in the arteries diminishes.

The velocity distribution and pressure profile are re-examined with application of a varying outlet pressure boundary condition, as shown in Fig. 4. Figures S3 and S4 show the velocity distribution in the aorta comparing the application of the two differing outlet pressure boundary conditions. These figures depict the velocity distribution prior to stent deployment as well as after stent deployment against three different flap stiffness configurations (1:1, 1:0.1, 1:0.01). The application of an oscillating pressure boundary condition does not significantly impact the velocity distribution observed across the cardiac cycle. Figures S5 and S6 similarly show the pressure distribution in the aorta across systole and diastole both with a constant pressure boundary condition and with a varying pressure boundary condition. In Fig. S5, the magnitudes of the achieved pressures at systole are elevated in the simulations with a varying pressure outlet boundary condition as compared to the constant pressure outlet boundary condition. However, the evolved pressure gradient is similar irrespective of applied boundary condition. Figure S6 demonstrates similar convergence between simulations irrespective of boundary condition definition, however in this case the pressure values are lower at diastole in the varying pressure outlet boundary condition simulations as compared to the constant pressure outlet boundary condition simulations.

## Discussion

As detailed previously, although hemiarch repair remains favored due to relatively lower neurologic morbidity compared to TAR, the residual dissection flap creates an avenue for further aortic degeneration. Our *in-silico* analysis of BMS in a patient-specific geometry demonstrates its viability as an adjunct to stabilize the aortic arch and manage the residual dissection flap. The Cook Zenith Dissection Stent system was designed to incorporate a proximal covered stent portion in the thoracic aorta with a distal bare metal component in the abdominal aorta. The presence of the great vessels precludes simply shifting this system proximally for our purpose. In this paper, we show that the BMS can provide a similarly stabilizing effect to the residual flap after hemiarch, like how it stabilizes the abdominal aorta when utilized in its intended fashion.

Our computational results suggests that this stabilizing effect is achieved via positive aortic remodeling and creation of smoother velocity profile and arch de-pressurization. These results are particularly notable when we note that the low radial force of the chosen stent, as reported in the literature, is insufficient to achieve complete intimal flap apposition to the aortic wall in even the most acute setting for this patient geometry. Figures 9 and 10 demonstrate positive aortic remodeling, clinically thought of as true lumen expansion with accompanying false lumen contraction. Additionally, Figs. 11 and 12 together demonstrate an improved hemodynamic profile of the aortic arch with a smoother velocity profile and a depressurized arch. Figures S3-S6 demonstrate that the smooth velocity profile and depressurized arch are maintained when an oscillating outlet pressure boundary condition is applied. This supports our result that the smoothing of the pressure gradient is not simply a consequence of the arbitrarily chosen uniform outlet pressure boundary condition in Fig. 12. When considering the hemodynamic profile of the aorta post-BMS, it is important to note that our clinical repair does allow for continued flow into the false lumen via the proximal tear. Despite this continued opportunity for flow, we still demonstrate a reduction in the false lumen volume as well as a decreased pressure gradient in the proximal aorta. Taken together, these simulation findings suggest bare metal stenting of the arch can assist in reconstituting the hemodynamics of the native un-dissected aorta thereby preventing further aortic degeneration associated with a persistent dissection flap and pressurized false lumen.

The impact of the ratio of aorta-to-flap stiffness was profound. It is notable that even with an aorta-to-flap ratio of 1:0.01 there was still a persistent false lumen. Nevertheless, as Figs. 12, S5 and S6 attest, we were still able to achieve depressurization of the aortic arch in this setting. The technical implications of this finding are significant. Clinically there is concern that ballooning a stent in the aortic arch against a stiffer (assumed to be more chronic) flap carries risk. Our simulation suggests that even achieving a technical result here that is less than ideal (with ideal defined as total elimination of the false lumen) does result in positive hemodynamic changes in the diseased aorta. As aneurysmal degeneration is critical to avoid in those with a residual dissection flap, it is important to appreciate that total false lumen elimination is simply a surrogate goal with the primary one being lowering of intra-aortic blood pressure. The sensitivity of the hemodynamic impact of stenting to flap stiffness also suggests need for greater research into the utility of technologies such as optical coherence tomography or intravascular ultrasound to improve flap characterization. Such technologies may allow more precise quantification of the risks of ballooning in the aortic arch.

The pressure distribution highlighted in Figs. 12, S5, and S6 corresponding to the unstented aorta hints at an underappreciated physiologic consequence of residual dissection flaps—dramatic pressure differentials across the aorta. This pressure distribution can be thought of as akin to a functional coarctation physiology. Currently, the risk for aneurysmal degeneration in patients having undergone hemiarch repair is conceptualized as stemming from a material defect in the residual flap. Our simulation results suggest that the coarctation-like physiology imposed by the presence of the residual flap maybe another driving factor in negative outcomes. It is notable that a larger increase in the diameter of the true lumen after stenting decreases both the pressure in the proximal aorta (which has already been through a hemiarch repair) as well as the pressure gradient along the aorta. Our results posit that the presence of the residual dissection flap may itself worsen the hemodynamics of the aorta by escalating the pressure in the proximal aorta thereby further increasing the risk of aneurysmal degeneration. This may provide a possible explanation for why patients with residual dissection flaps experience such deleterious outcomes despite universal counseling on the need for blood pressure control. BMS can be thought of as mechanistically breaking this self-perpetuating cycle of worsening blood pressure gradients in the aorta leading to degeneration in patients with a residual dissection flap.

Finally, it is worth noting the utility of our FEA-based approach to understand the evolution of aortic pathology and disease management. Given that aneurysmal and dissection pathology is a fundamentally mechanics driven process, analysis of stress focusing and aortic displacement via FEA has a basis in the literature [20, 38]. The relative fidelity of our stent deployment as compared to angiographic evidence is consistent with prior literature [24, 26, 39]. We hope this paper contributes towards the existing body of research arguing for the utility of a computational approach incorporating FEA as a valuable tool to gain surgical insight and understand vascular biomechanics. In this case, our analysis can be thought of as a feasibility study regarding the performance and suitability of the Zenith Dissection Endovascular Stent in an aortic region outside of IFU. A similar translation from a computational domain to a surgical operating room has been done in pediatric cardiac surgery research, specifically utilizing CFD to optimize treatment of single-ventricle pathology and better understand the biomechanical impacts of surgeries for this condition [40]. Given that our analysis is performed on a single case, we will of course require further work before widespread incorporation. We hope that for patients in difficult situations, such as those who are not candidates for TAR this may provide an avenue for managing the residual dissection flap after a hemiarch. Figure 7 attests to the adaptability of the Zenith device to the aortic arch as it demonstrates relatively minimal stress focusing. Given the important role that well-intentioned off-label usage of endovascular devices plays in device development our approach of FEA and in silico investigation of device suitability is valuable in investigating the suitability of devices for off-label uses.

## Limitations

This is a computational feasibility analysis on a single patient thus limiting its generalizability. We view these results as hypothesis generating and will require greater clinical study prior to widespread incorporation. Our approach clinical approach has several limitations including the BMS allowing for continued flow into the false lumen given it is not covered. In addition, the use of a BMS allows for further flow between the true and false lumen if there are any re-entry tears.

With regards to our computational modeling there are several limitations. The segmentation of the flap is currently a challenging process that does rely upon user definition of the false and true lumens. Given resolution issues we cannot directly always visualize the flap at all slices of a CT scan. As such, we assume a flap that does not have any re-entry tears. Our flap-stiffness ratios are sourced from literature and not patient-specific. Our CFD boundary conditions are not patient-specific, which potentially limits applicability of our results. Our aorta is also modelled utilizing a rigid-wall assumption in our CFD. The lack of compliance may influence the development of pressure gradients which we analyzed. Furthermore, we did not have access to a post-operative geometry with the same configuration, of hemiarch followed by BMS, given that the patient subsequently underwent TEVAR. As such, we cannot validate the CFD results against a corresponding post-operative patient-specific geometry.

## Conclusion

Hemiarch repair offers a relatively safer approach to type A dissection management than TAR however it leaves a residual dissection flap that poses a clinical challenge. Amongst the armamentarium of evolving options, we demonstrate that bare metal stenting is an important adjunct to hemiarch repair. Our FEA and CFD analysis of a patient-specific geometry shows how BMS improves the hemodynamics of the dissected aorta after hemiarch repair illustrating a mechanistic motivation for further adoption of this surgical technique.

## Supplementary Information

Below is the link to the electronic supplementary material.

Supplementary file1 (DOCX 4848 KB)

## Data Availability

Data will be placed into repository regarding simulations.
