# Supplementary material for: Bare Metal Stenting for Residual Arch Dissections: A Computational Analysis
Source: Cardiovasc Eng Technol. 2025 Aug 4;16(6):624–40. doi: 10.1007/s13239-025-00799-6 (PMC12686075; doi:10.1007/s13239-025-00799-6)
Supplement: Supplementary file 1 — Supplementary file1 (DOCX 4848 KB) [file 13239_2025_799_MOESM1_ESM.docx]

**Supplemental Figures**


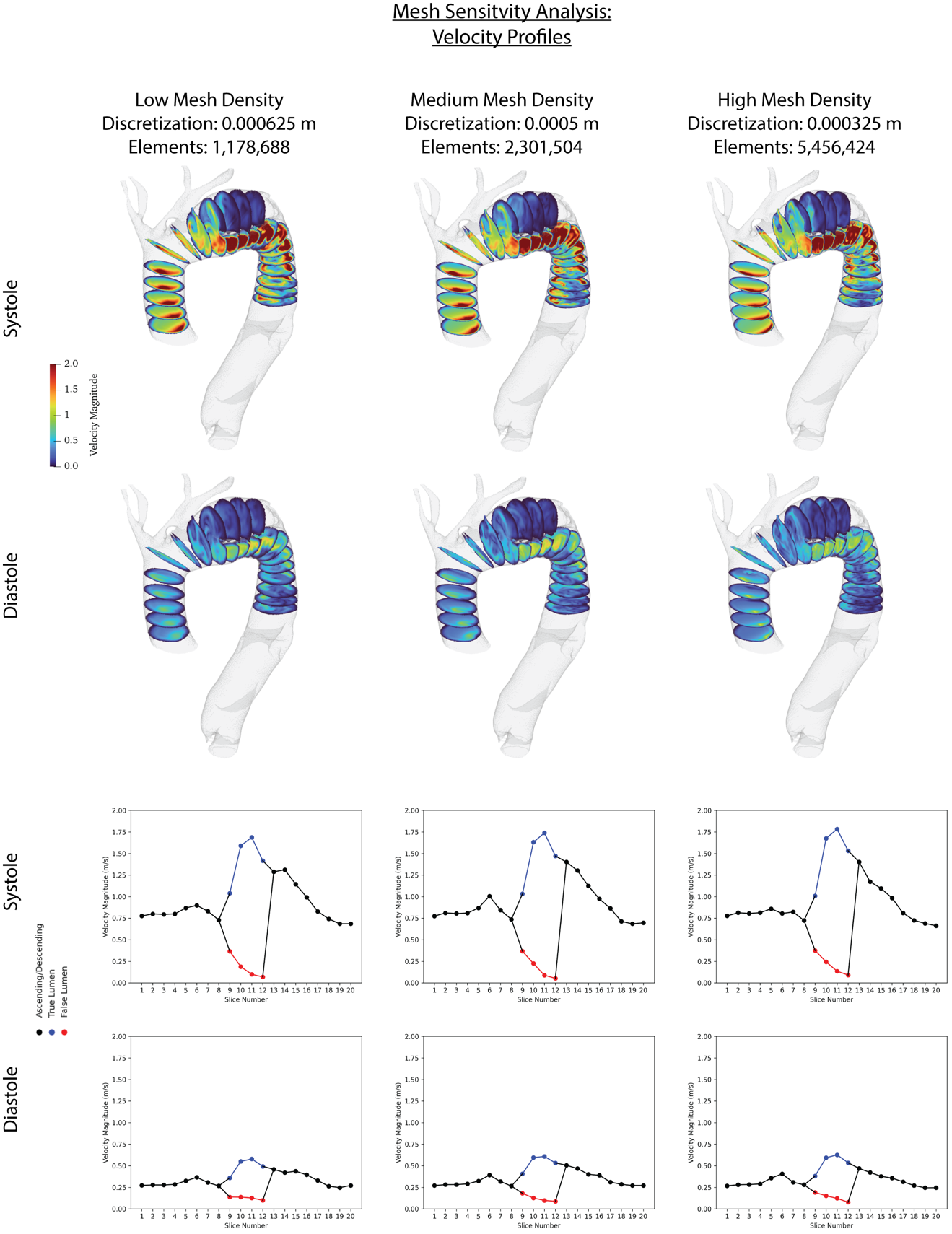


Supplemental Figure S1: Velocity distribution in aortas at varying mesh densities in both systole and diastole.


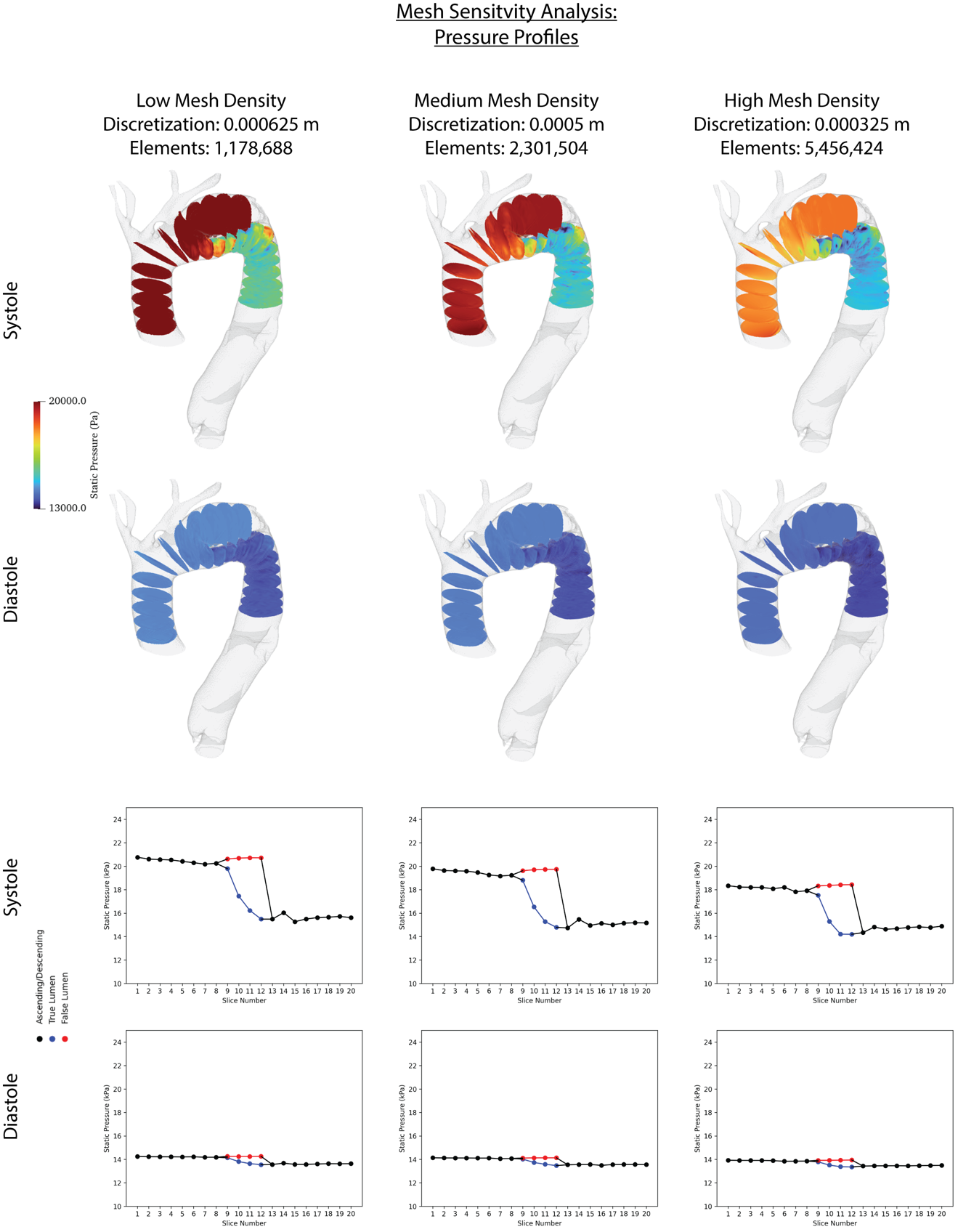


Supplemental Figure S2: Pressure Distribution in aortas at varying mesh densities in both systole and diastole.


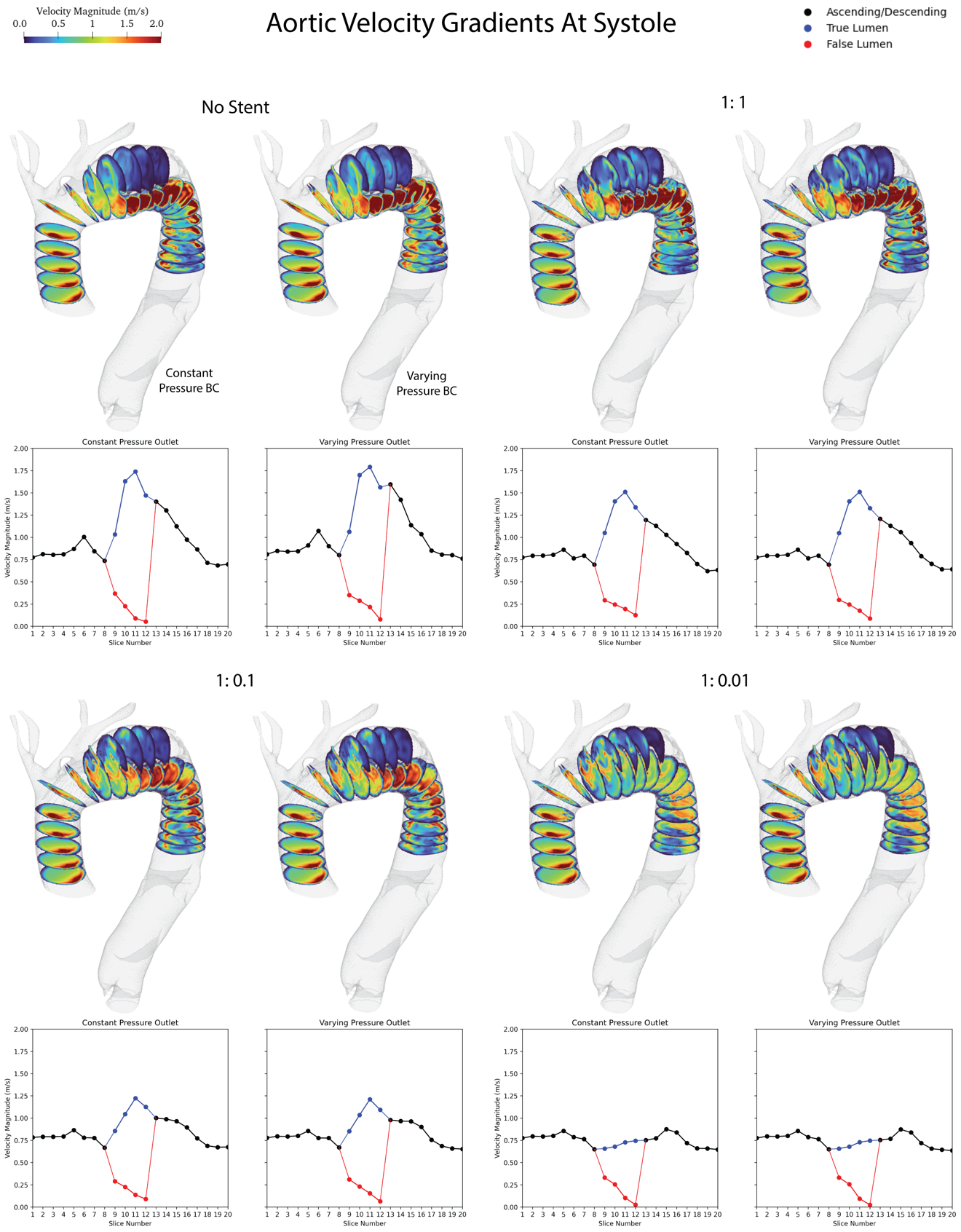


Supplemental Figure S3: Fluid velocity throughout the aorta in systole in non-stented and three different flap stiffness configurations (1:1, 1:0.1, and 1:0.01). The bulk fluid velocity is shown in 20 slices along the lumen, where true and false lumen are separated. For each configuration, simulations results are shown for constant pressure and varying pressure boundary conditions.


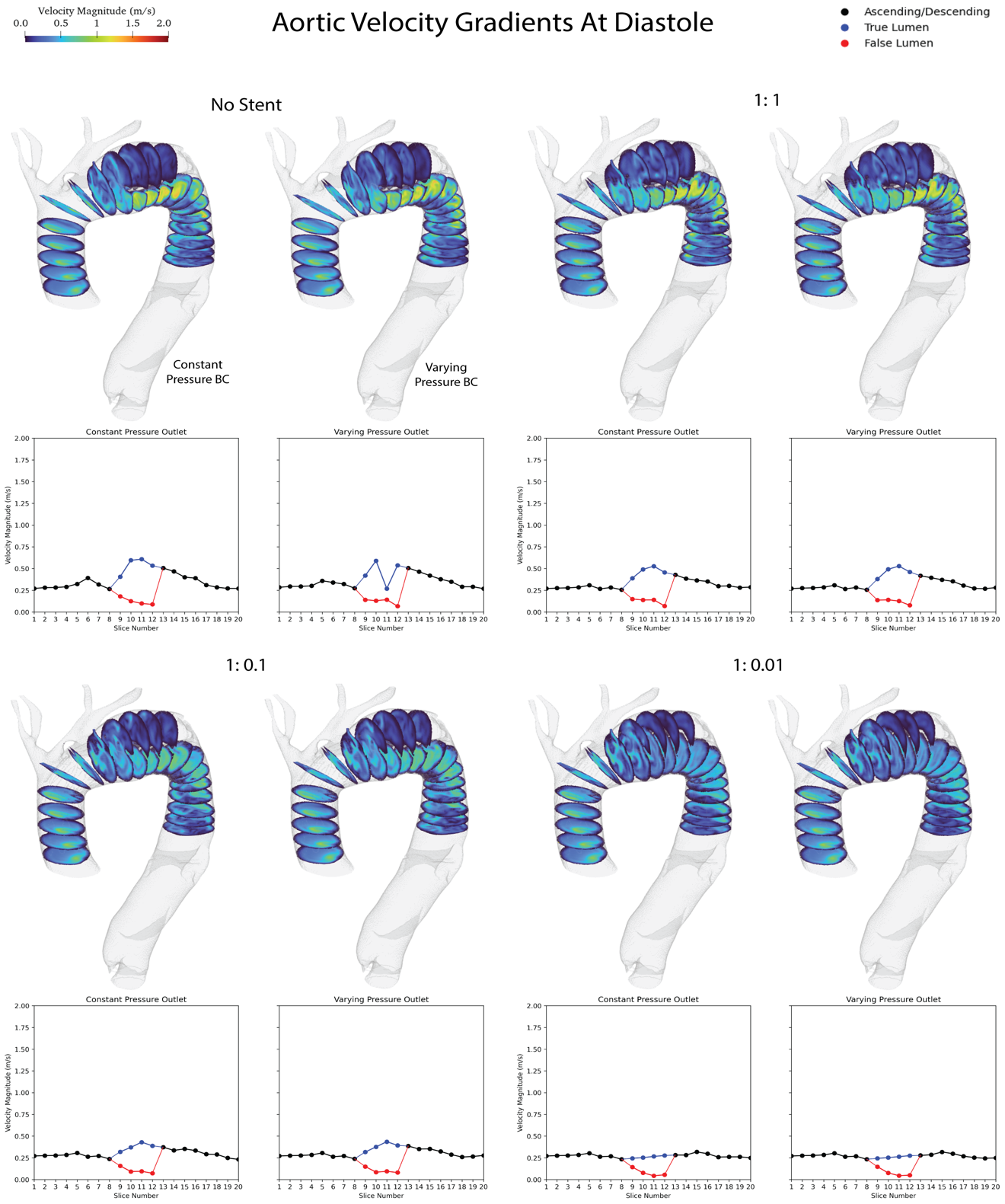


Supplemental Figure S4: Fluid velocity throughout the aorta in diastole in non-stented and three different flap stiffness configurations (1:1, 1:0.1, and 1:0.01). The bulk fluid velocity is shown in 20 slices along the lumen, where true and false lumen are separated. For each configuration, simulations results are shown for constant pressure and varying pressure boundary conditions.


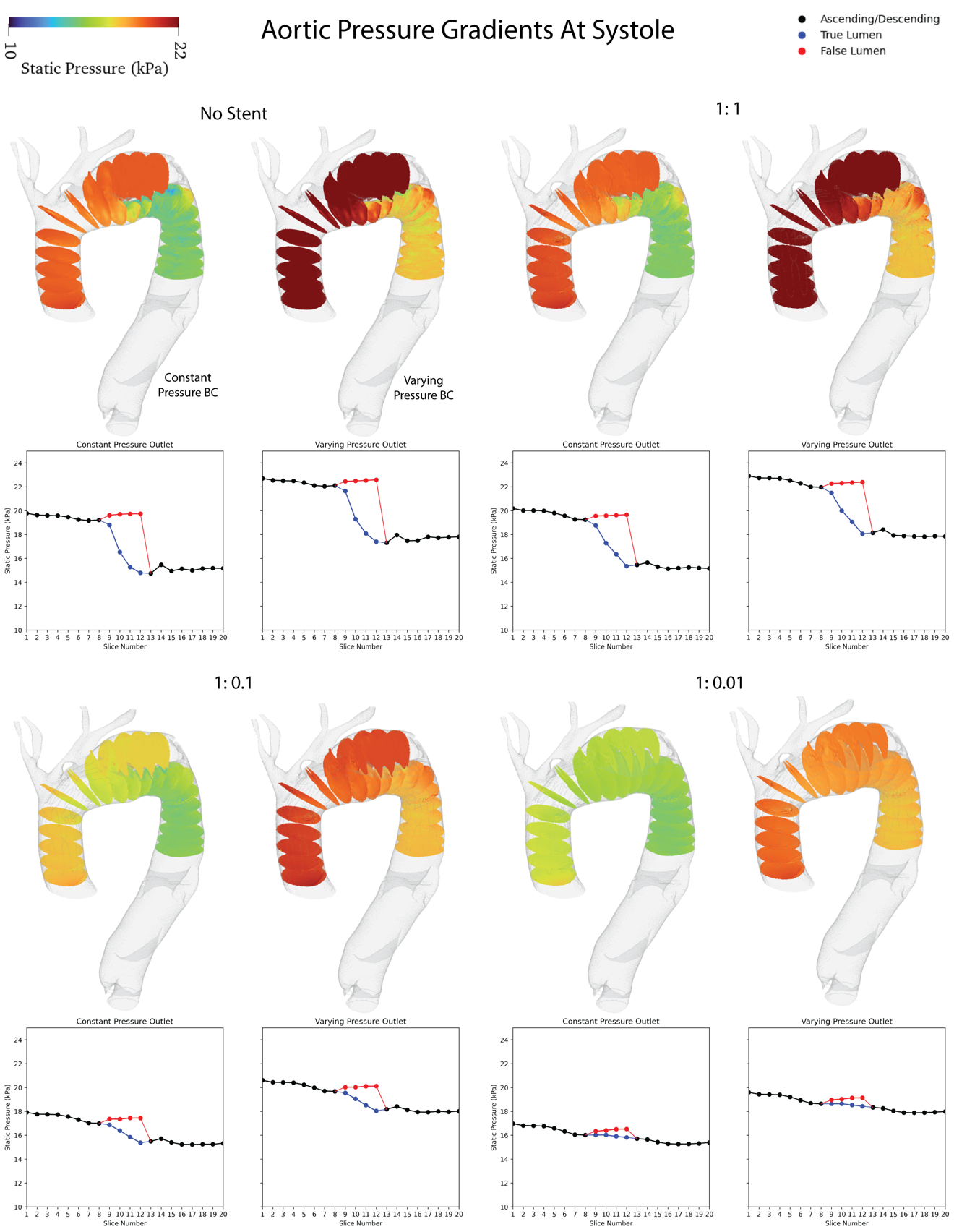


Supplemental Figure S5: Static pressure throughout the aorta in systole in non-stented and three different flap stiffness configurations (1:1, 1:0.1, and 1:0.01). The static pressure is shown in 20 slices along the lumen, where true and false lumen are separated. For each configuration, simulations results are shown for constant pressure and varying pressure outlet boundary conditions.


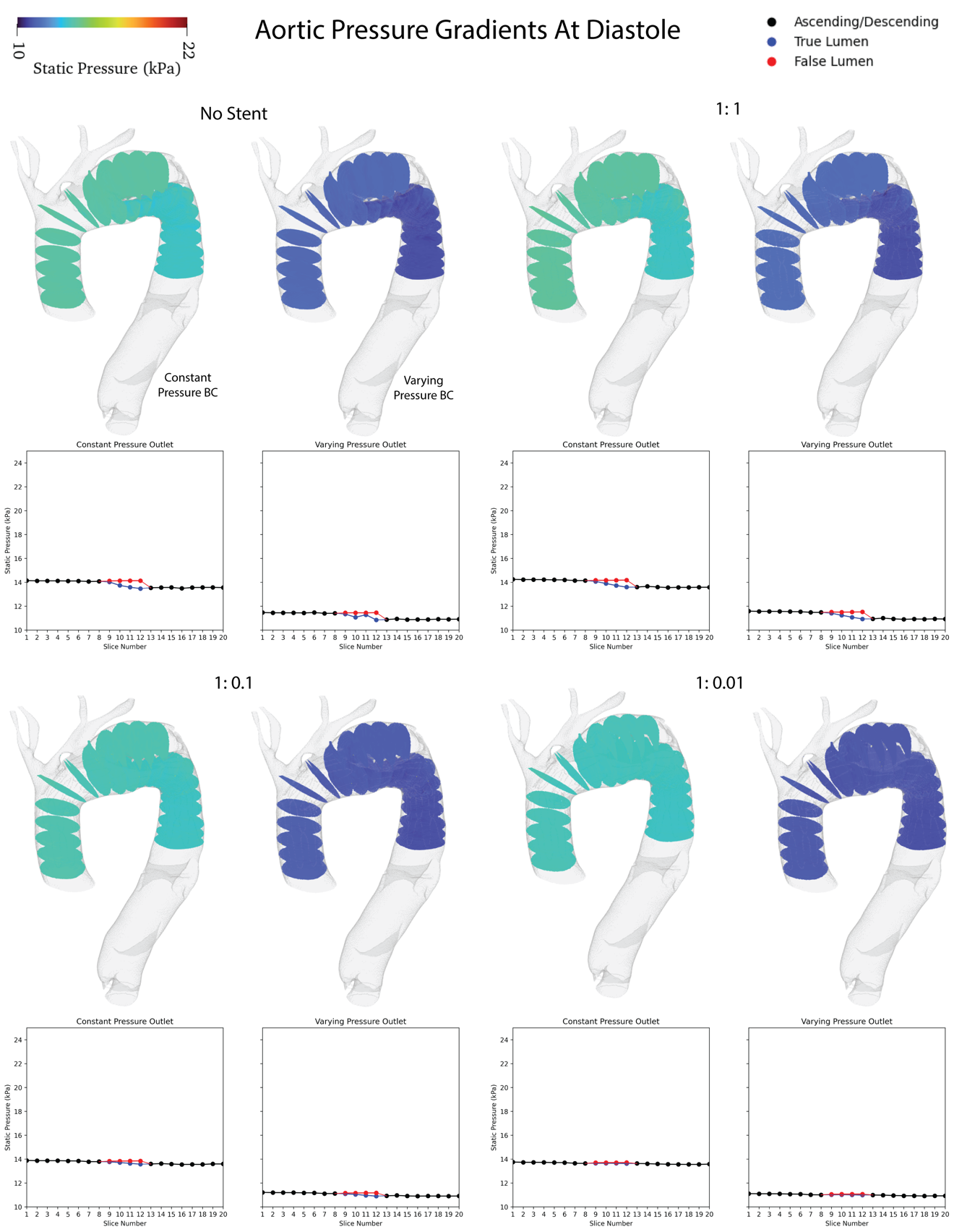


Supplemental Figure S6: Static pressure throughout the aorta in diastole in non-stented and three different flap stiffness configurations (1:1, 1:0.1, and 1:0.01). The static pressure is shown in 20 slices along the lumen, where true and false lumen are separated. For each configuration, simulations results are shown for constant pressure and varying pressure outlet boundary conditions.
